# The Role of Pyrrolidine in Antibacterial Drug Discovery: Clinically Approved Antibiotics, Novel Derivatives, and Future Perspectives

**DOI:** 10.3390/ijms27167225

**Published:** 2026-08-13

**Authors:** Aura Rusu, Ioana-Maria Stroia, Gabriel Hancu, Corneliu Tanase, Livia Uncu

**Affiliations:** 1 Pharmaceutical and Therapeutic Chemistry Department, Faculty of Pharmacy, George Emil Palade University of Medicine, Pharmacy, Science and Technology of Târgu Mureș, 540142 Târgu Mureș, Romania; aura.rusu@umfst.ro (A.R.); gabriel.hancu@umfst.ro (G.H.); 2 Pharmaceutical Botany Department, Faculty of Pharmacy, George Emil Palade University of Medicine, Pharmacy, Science and Technology of Târgu Mureș, 540142 Târgu Mureș, Romania; 3 Scientific Centre for Drug Research, Pharmaceutical and Toxicological Chemistry Department, “Nicolae Testemitanu” State University of Medicine and Pharmacy, 165 Bd. Stefan Cel Mare si Sfant, MD-2004 Chisinau, Moldova; livia.uncu@usmf.md

**Keywords:** pyrrolidine, pyrrolidine derivatives, pyrrolidine-based compounds, substituted pyrrolidine, heterocycles, five-membered heterocycles, antibiotics, antibacterials, antibacterial activity, nitrogen heterocycles

## Abstract

Antimicrobial resistance is a major global health challenge that has created an urgent need for new antibacterial agents capable of overcoming emerging resistance mechanisms. Among nitrogen-containing heterocycles, pyrrolidine has been widely investigated in medicinal chemistry due to its structural versatility, favourable physicochemical properties, and ability to enhance interactions with biological targets. This review provides a comprehensive and critical overview of pyrrolidine-based compounds investigated for antibacterial applications. Relevant studies describing clinically approved antibiotics, natural products, synthetic derivatives, hybrid molecules, and antibacterial adjuvants containing a pyrrolidine scaffold were collected, classified, and critically evaluated, with particular emphasis on structural features, antibacterial activity, and structure–activity relationships. The reviewed evidence demonstrates that the pyrrolidine moiety is present in several antibacterial drug classes, including carbapenems, cephalosporins, fluoroquinolones, lincosamides, streptogramins, and tetracyclines, where it contributes to improved target affinity, antibacterial potency, and pharmacokinetic behaviour. Numerous recently reported pyrrolidine derivatives have shown promising activity against clinically relevant, multidrug-resistant bacterial pathogens. The pyrrolidine scaffold is valuable for the design of next-generation antibacterial agents and resistance-modifying compounds, though further in vivo studies and pharmacological evaluation are required to support their clinical development.

## 1. Introduction

### 1.1. Global Challenge of Antibacterial Resistance

According to the World Health Organisation (WHO), antibacterial resistance poses a significant health threat, causing over 1.27 million direct deaths annually by diminishing the efficacy of common antibiotics against widespread antibacterial infections. This global health crisis is projected to cause approximately 10 million deaths annually by 2050 [1,2]. Bacteria can develop resistance through various mechanisms, including genetic mutations and the transfer of resistance genes. As resistance continues to increase, the need for new antibacterial agents becomes more urgent, despite declining investment in antibiotic discovery and development [3,4].

Antimicrobial resistance is accelerated by inappropriate antibiotic prescribing, including antibiotic use for viral infections, unnecessary broad-spectrum therapy, incorrect dosing and duration, and extensive antibiotic use in human and veterinary medicine. One major challenge in addressing antibacterial resistance is understanding the true extent of resistance, particularly in regions with limited surveillance capacity [5]. Antimicrobial resistance is a priority challenge for public health specialists due to its widespread epidemiological implications. At the same time, health professionals are working to understand the mechanisms underlying bacterial resistance and to accelerate the development of new antibacterial molecules [6].

### 1.2. Heterocyclic Compounds in Medicinal Chemistry

Heterocyclic compounds represent a key class in medicinal chemistry due to their structural diversity and biological versatility, enabling effective interaction with biological targets [7]. Consequently, heterocycles are widely used as core scaffolds in the design of novel antibacterial agents [8].

Heterocyclic compounds are essential in drug design because they interact with biological targets and modulate key pharmacokinetic and pharmacodynamic properties. Among them, nitrogen-containing heterocycles have attracted significant attention due to their broad applications in biology, organic synthesis, and drug development [7,9,10,11]. Many heterocyclic compounds originate from natural products and have been optimised by medicinal chemists to improve their pharmacological properties. Nitrogen-containing heterocycles are particularly important as valuable scaffolds in drug discovery, including pyrrolidine, which can form favourable interactions with biological targets [12,13].

Five-membered heterocycles containing two or three heteroatoms are essential structural components of several antibacterials used to treat bacterial infections caused by various pathogens [14]. Therefore, heterocyclic nuclei are successfully used as bioisostere replacements of various structural fragments to improve the drug’s affinity for biological targets or reduce its toxicity [9,15].

### 1.3. Chemistry of Pyrrolidine

**Figure 1:**
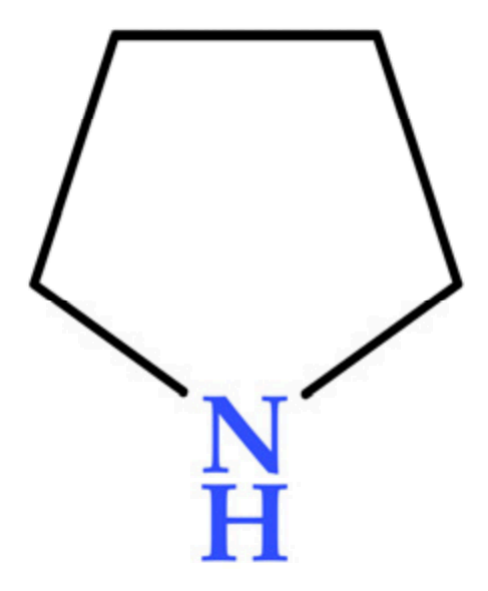
Molecular structure of pyrrolidine.

Pyrrolidine is the first saturated member of the family of five-membered heterocycles with one nitrogen atom. Its molecular structure is shown in Figure 1.

Since the chemistry and synthetic methodologies of pyrrolidine have already been reviewed in the literature [16,17,18], only the key chemical properties relevant to antibacterial medicinal chemistry are briefly summarised here. The heterocycle, commonly known as tetrahydropyrrole, is categorised as an aza-cycloalkane (aza-cyclopentane), a type of cyclic amine. Unsubstituted pyrrolidine readily undergoes N-alkylation, whereas appropriately substituted derivatives may undergo acylation, nitrosation, or other reactions characteristic of cyclic amines. Pyrrolidine and *N*-substituted pyrrolidines exhibit chemical reactivity similar to that of secondary and tertiary alkylamines. As cyclic amines, they readily undergo the characteristic reactions of these amine classes [17,19].

The pyrrolidine ring is a relatively flexible, non-planar, polar heterocycle due to the presence of its amine nitrogen. Its basicity is higher (p*K*_a_ 11.3) than that of the acyclic compound diethylamine (p*K*_a_ 10.49). The secondary amine nitrogen confers basic character on pyrrolidine and is predominantly protonated under physiological conditions. Consequently, incorporation of a pyrrolidine ring may increase aqueous solubility and enable ionic interactions with biological targets, although the overall effect depends on the complete molecular structure [17,20]. In pyrrolidine, the cyclic framework restricts the orientation of the ring carbon atoms, leaving the nitrogen lone pair more accessible to electrophilic attack. Consequently, pyrrolidine is a more effective nucleophile than diethylamine, in which conformational flexibility can generate steric hindrance around the nitrogen atom [18].

Unlike traditional flat aromatic rings, the pyrrolidine scaffold has an sp3-rich hybridisation and a non-planar heterocycle, providing enhanced three-dimensional spatial coverage [16]. Unlike pyrrole, whose aromatic structure limits stereochemical diversity, pyrrolidine can have up to 4 stereogenic carbons, yielding up to 16 stereoisomers. Since different stereoisomers may display distinct pharmacological profiles, the development of stereoselective synthetic approaches capable of generating pyrrolidines with defined configurations is essential for the discovery and optimisation of new drug candidates [16,21].

Simultaneously, pyrrolidine undergoes pseudorotation, an intrinsic conformational process of saturated five-membered rings. Hence, pyrrolidines adopt energetically favourable conformations that provide 3D coverage, a useful tool for exploring pharmacophore space via diversity-oriented synthesis (DOS) [22,23].

### 1.4. Pyrrolidine as a Valuable Scaffold in Medicinal Chemistry

Firstly, pyrrolidine scaffolds are used as heterocyclic structures in medicinal chemistry, appearing in numerous U.S. Food and Drug Administration (FDA)-approved drugs and active molecules due to their sp3-rich, non-planar structures, which enhance 3D pharmacological properties [24].

Secondly, pyrrolidine has attracted the attention of medicinal chemists for several reasons: chiral pyrrolidines have become leading scaffolds in ligand design, whether in transition-metal-mediated or organocatalytic processes; many substituted pyrrolidine derivatives exhibit a wide range of bioactivities, including antibiotic, antifungal, antiviral, anti-inflammatory, and cytotoxic effects. Lastly, the pyrrolidine ring constitutes the central structure of proline, a compound with one chiral centre, which is frequently used as a building block for the synthesis of chiral compounds. All these favourable properties offer an excellent opportunity to discover new pharmaceutical agents [16,21,25].

The pyrrolidine ring is widely recognised as a valuable scaffold in medicinal chemistry and antibacterial drug design [12,16,26].

In antibacterial medicinal chemistry, pyrrolidine can be used as a pharmacophore, a conformationally constrained linker, or an ionisable substituent. Depending on its substitution and position within the molecular architecture, it may modulate target recognition, solubility, bacterial penetration, and pharmacokinetic behaviour. Pyrrolidine-containing antibiotics consequently act through the mechanism of their respective pharmacophores, including inhibition of cell-wall biosynthesis, bacterial DNA replication, or ribosomal protein synthesis; the pyrrolidine moiety primarily optimises these interactions rather than determining a single antibacterial mechanism of its own [7,9,12,13].

Pyrrolidine is already a validated antibacterial scaffold because it occurs in multiple clinically successful antibiotics. This success has stimulated the design of numerous new antibacterial derivatives. More details regarding the role of pyrrolidine in the structure of certain antibiotics can be found in Section 2.

### 1.5. Need for New Pyrrolidine-Based Antibacterial Agents

While the current clinical armamentarium includes several highly effective antibacterial drugs, their long-term efficacy is severely affected by the continuous and fast evolution of antibacterial resistance, especially by a series of bacteria known as extremely resistant—*Enterococcus faecium*, *Staphylococcus aureus*, *Klebsiella pneumoniae*, *Acinetobacter baumannii*, *Pseudomonas aeruginosa*, and *Enterobacter* sp. The listed bacteria are known as the ESKAPE pathogens, which share adaptations that enable them to survive in modern health-care settings [27].

A suboptimal safety profile, including poor pharmacokinetics or high target toxicity, frequently hinders the clinical application of novel antibiotics. Thanks to its favourable properties, the pyrrolidine ring offers medicinal chemists a versatile platform for balancing antibacterial potency with physicochemical and pharmacokinetic properties, while minimising adverse effects. By developing next-generation pyrrolidine derivatives, current medicinal-chemistry efforts aim to achieve superior target binding affinities and an improved pharmacokinetic profile.

Several FDA-approved antibacterial agents incorporate a pyrrolidine or pyrrolidinium moiety into their pharmacologically relevant molecular architecture. Representative examples are summarised in Table 1.

### 1.6. Rationale of the Present Study

The rapid emergence of antimicrobial resistance significantly limits the effectiveness of existing antibiotics, largely due to mechanisms such as target-site modification and efflux pump overexpression [38]. Consequently, the exploration of new chemical scaffolds has become a critical priority in antibacterial drug discovery [39,40].

Pyrrolidine-based heterocycles represent a promising structural class, owing to their favourable physicochemical and pharmacological properties. Therefore, the design and development of novel pyrrolidine-derived compounds may offer valuable opportunities to identify new antibacterial agents capable of overcoming current resistance mechanisms. Although several recent reviews [41,42] have discussed antibacterial heterocycles, individual antibiotic classes, pyrrolidine-based hybrid molecules, or patented pyrrolidine derivatives with antibacterial activity, an updated analysis of the role of the pyrrolidine scaffold across clinically approved antibiotics, investigational antibacterial candidates, hybrid molecules, and antibacterial adjuvants is necessary. Recently, several important advances were published, including newly approved or clinically updated pyrrolidine-containing antibiotics and recently reported antibacterial candidates. This review was therefore designed to fill this knowledge gap by providing an integrated analysis of the structural, pharmacological, and medicinal chemistry aspects of pyrrolidine-containing antibacterial compounds. The novelty of the present review lies in its comprehensive analysis of the pyrrolidine scaffold across approved antibiotics, antibacterial adjuvants, natural products, and recently reported antibacterial candidates.

This review provides an updated, critical analysis of pyrrolidine-containing compounds in antibacterial drug discovery. It integrates clinically approved antibiotics, natural and synthetic antibacterial candidates, hybrid molecules, and resistance-modifying adjuvants, with particular emphasis on their structural features, mechanisms of action, and structure–activity relationships.

## 2. Pyrrolidine Five-Membered Heterocycle as a Scaffold in Medicinal Chemistry

### 2.1. Pyrrolidine Derivatives with Biological Properties

The pyrrolidine ring is a ubiquitous structural motif in nature, prominently featured in the structure of the essential amino acid proline, which serves as a fundamental building block for numerous peptides and proteins. In addition to its structural role in biology, the pyrrolidine nucleus is considered a valuable pharmacophore in medicinal chemistry [16,20].

Its widespread presence in bioactive compounds is directly attributed to its unique physicochemical properties. The pyrrolidine nucleus is highly valued for its non-planar structure and sp3-hybridised nature, which allows it to explore three-dimensional pharmacophore space. Given that the secondary amine of the pyrrolidine ring is predominantly protonated at physiological pH. This aspect allows the molecule to form strong ionic and hydrogen-bonding interactions with complementary amino acid residues in the biological receptor and the active sites of enzymes [12,16,17,24]. Also, the cyclic structure of the ring offers an optimal balance between flexibility and conformational rigidity, which enhances target affinity while improving aqueous solubility and overall pharmacokinetic profiles [16,18]. Thus, pyrrolidine derivatives exhibit an exceptionally broad spectrum of pharmacological activities and have been widely investigated and developed as potent antineoplastic, anti-inflammatory, antibacterial, antifungal, and antiviral agents [24,43].

Pyrrolidine is present in the structures of natural alkaloids, such as nicotine, hygrine, cuscohygrine, and atropinone, and the most important amino acid that contains a pyrrolidine heterocycle is proline. Bioactive compounds that contain a pyrrolidine scaffold continue to be studied for the development of new pharmacologically active candidates. Certain pyrrolidine derivatives have been identified as pharmacophoric motifs associated with antibacterial activity [20,24].

#### 2.1.1. Natural Compounds Containing a Pyrrolidine Heterocycle

Nature is a rich source of pyrrolidine-containing molecules, providing basic compounds of immense value for drug discovery. These naturally occurring products exhibit potent, interesting, and various biological activities [20,44]. Natural products containing a pyrrolidine moiety have attracted considerable attention owing to their structural complexity and diverse biological properties. The structures of several natural alkaloids, like amathaspiramide A, B and C, atropine, bgugaine, irniine, cocaine, codonopsinine and codonopsine, ficushispimine A and B, hygrine, nicotine, radicamine A and B, scalusamide A and many more, have a pyrrolidine ring in their structure [20,44,45].

The pyrrolidine ring is an important biosynthetic building block in the formation of biologically active alkaloids, including atropine, hyoscyamine, scopolamine, cocaine, and nicotine, which exert diverse effects on the nervous system [20,44,46,47]. Polyhydroxy alkaloids, such as radicamines A and B, isolated from *Lobelia chinensis*, were studied for their antidiabetic potential, specifically their α-glucosidase inhibitory activity [48]. Other polyhydroxy pyrrolidine alkaloids, codonopsine and codonopsinine, isolated from *Codonopsis clematidea*, serve as structural templates for the design of antibacterial agents; specifically, the codonopsinine analogue MFM 501 exhibits antibacterial activity against methicillin-resistant *Staphylococcus aureus* (MRSA) [45].

Terrestrial and marine environments have also given complex pyrrolidine derivatives with notable anti-infective and cytotoxic properties. Alkaloids such as bgugaine and irniine isolated from *Arisarum vulgare* exhibit strong antibacterial, antifungal, and cytotoxic properties [44]. Ficushispimines A and B similarly exhibit antibacterial activity. From the marine environment, amathaspiramides A, B, and C present potent antiviral and cytotoxic effects. At the same time, scalusamides A, B, and C, isolated from the marine fungus *Penicillium citrinum*, demonstrated antifungal and antibacterial activity against pathogens, including *Micrococcus luteus* and *Cryptococcus neoformans* [44,49,50].

Marine and aquatic organisms produce pyrrolidine-containing metabolites, including kainic acid and preussin, with kainic acid serving as a powerful glutamate receptor agonist and an important tool in neuroscience research [26,51]. Also, among naturally occurring pyrrolidine derivatives, stachydrine has attracted considerable interest owing to its diverse therapeutic potential, particularly in cancer, cardiovascular, renal, and gynaecological disorders [52].

The compounds discussed in this section are presented to illustrate the occurrence and structural diversity of the pyrrolidine scaffold in natural products. Their inclusion provides a general medicinal chemistry context for understanding the widespread biological relevance of pyrrolidine-containing molecules. Natural compounds that have been specifically investigated for antibacterial activity are discussed in detail later in Section 3 within the context of pyrrolidine-based antibacterial candidates.

#### 2.1.2. Approved Antibiotics Containing a Pyrrolidine Heterocycle

As a pharmacophore group, pyrrolidine is found in the molecular structures of different classes of medicines, including an extensive series of antibiotics: beta-lactam antibiotics, lincosamides, fluoroquinolones, streptogramins, and tetracyclines (Table 2).

### 2.2. Overview of Approved Antibacterial Drug Classes from Chemical and Clinical Perspectives

#### 2.2.1. Beta-Lactam Antibiotics

1. Carbapenems

**Figure 2:**
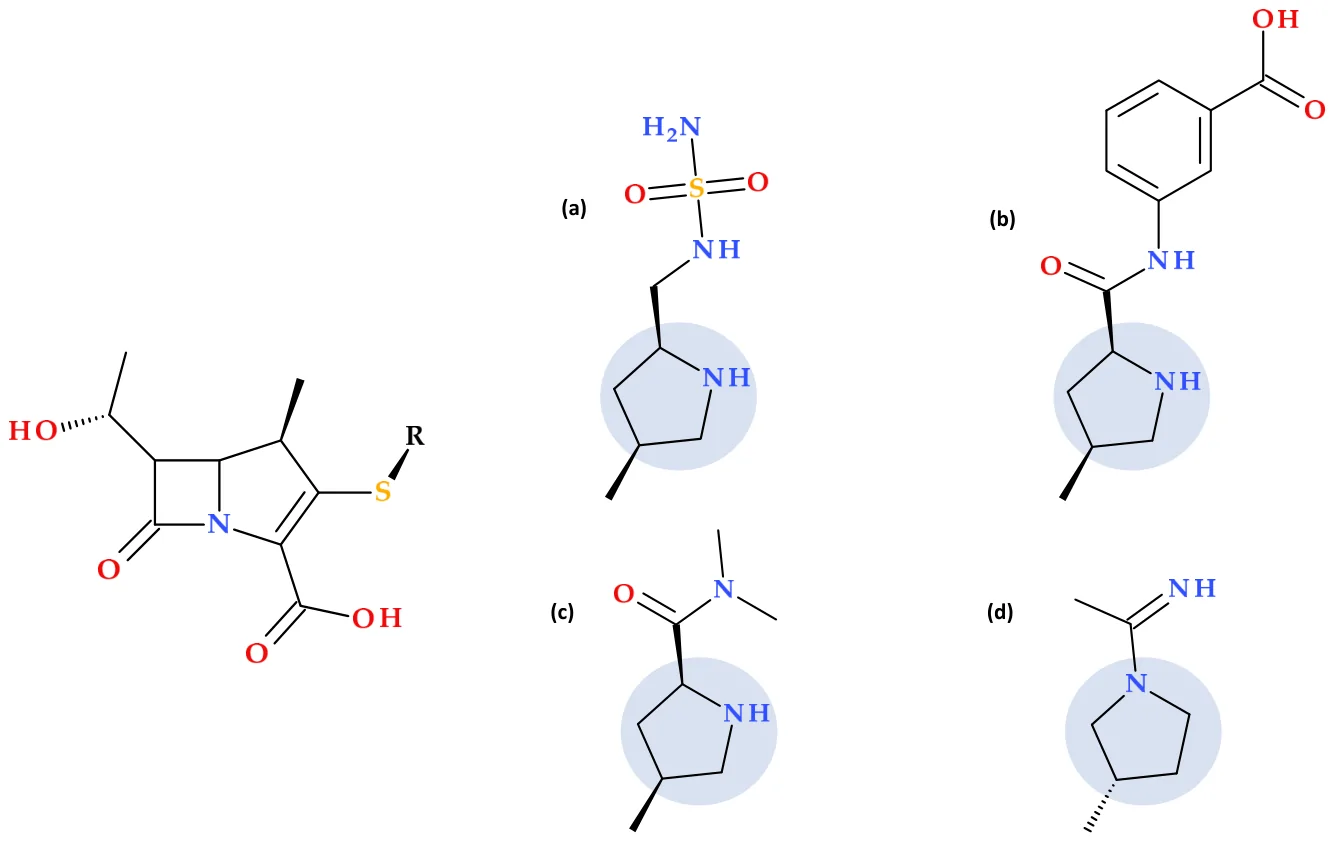
Molecular structures of pyrrolidine-based carbapenems, where R is for: (**a**) Doripenem, (**b**) Ertapenem, (**c**) Meropenem, and (**d**) Panipenem.

Carbapenems are a class of beta-lactam antibiotics characterised by potent activity against a wide range of Gram-positive and Gram-negative bacteria and exceptional stability against many beta-lactamases [53,63,64]. Doripenem, Ertapenem, Meropenem, and Panipenem are carbapenems that contain pyrrolidine-derived side chains (Figure 2) [64].

The introduction of a pyrrolidine moiety into the molecular structure of the four antibiotics is an essential modification that enhances both their antimicrobial spectrum and stability compared with earlier carbapenems. Among clinically available carbapenems, pyrrolidine-containing derivatives (such as Meropenem, Ertapenem, and Doripenem) (Figure 2) display broad activity against Gram-negative pathogens and are resistant to hydrolysis by many beta-lactamases. A pyrrolidine side chain, together with a 1-beta-methyl group, also improves stability against renal dehydropeptidase I (DHP-I), eliminating the need for coadministration of a DHP-I inhibitor [53,64,65].

Although structurally related, these carbapenems differ in spectrum. Meropenem exhibits excellent activity against a wide range of Gram-negative organisms and is more active than Imipenem against many Enterobacterales. Doripenem exhibits particularly strong activity against *Pseudomonas aeruginosa* and *Acinetobacter baumannii*, often yielding lower Minimum Inhibitory Concentration (MIC) values than those of Imipenem and meropenem. Ertapenem has a longer half-life and a narrower spectrum, with reduced activity against *Pseudomonas aeruginosa* and other non-fermenting Gram-negative bacteria [53,64].

Panipenem is a broad-spectrum beta-lactam antibiotic with activity against Gram-positive and Gram-negative bacteria, both aerobic and anaerobic. The compound contains a 3-acetamidoylpyrrolidinyl substitution and one methyl group at position C1 (Figure 2d). The antibiotic is administered parenterally and is considered more stable than Imipenem against hydrolysis by DHP-1; however, it remains susceptible to hydrolysis by renal DHP-1. Panipenem is administered with a DHP-1 inhibitor, such as Betamipron, in a 1:1 ratio [66,67]. Although Panipenem has been approved and marketed in Japan (Panipenem/Betamipron; Carbenin^®^), it has not been authorised by the FDA or the European Medicines Agency (EMA), resulting in limited international availability [54].

Clinical studies have shown that the combination of the two substances has high efficacy against respiratory and urinary tract infections (UTIs) in adults and is also effective in treating listerial meningitis in paediatric patients. Panipenem exhibited good antimicrobial activity against Gram-positive aerobic bacteria. Regarding antibacterial activity against Gram-negative aerobic bacteria, it was notable that most tested *Enterobacteriaceae* were susceptible to Panipenem, including *Klebsiella* sp., *Proteus* sp., *Enterobacter* sp., and *Escherichia coli* [67].

In summary, Doripenem, Ertapenem, and Meropenem are pyrrolidine-derived carbapenems whose pyrrolidine substituent confers enhanced stability, broad-spectrum antibacterial activity, and favourable pharmacological properties, making them important agents for the treatment of serious bacterial infections. While Doripenem, Ertapenem, and Meropenem are widely used in contemporary clinical practice, Panipenem remains approved primarily in Japan, where it is still used to treat severe bacterial infections. All these carbapenems represent important therapeutic options for the management of serious bacterial diseases.

2. Cephalosporins

Cephalosporins constitute a subclass of beta-lactam antibiotics in which the beta-lactam ring is fused to a six-membered dihydrothiazine ring, in contrast to the five-membered thiazolidine ring characteristic of penicillins. The basic 7-aminocephalosporanic acid skeleton was modified at multiple positions to yield compounds with improved antibacterial activity and optimised pharmacokinetic properties [66].

Pyrrolidine, as a fundamental structural element, is found in various cephalosporins, conferring valuable properties, such as stability against beta-lactamases and an expanded spectrum of activity (Table 2).

**Figure 3:**
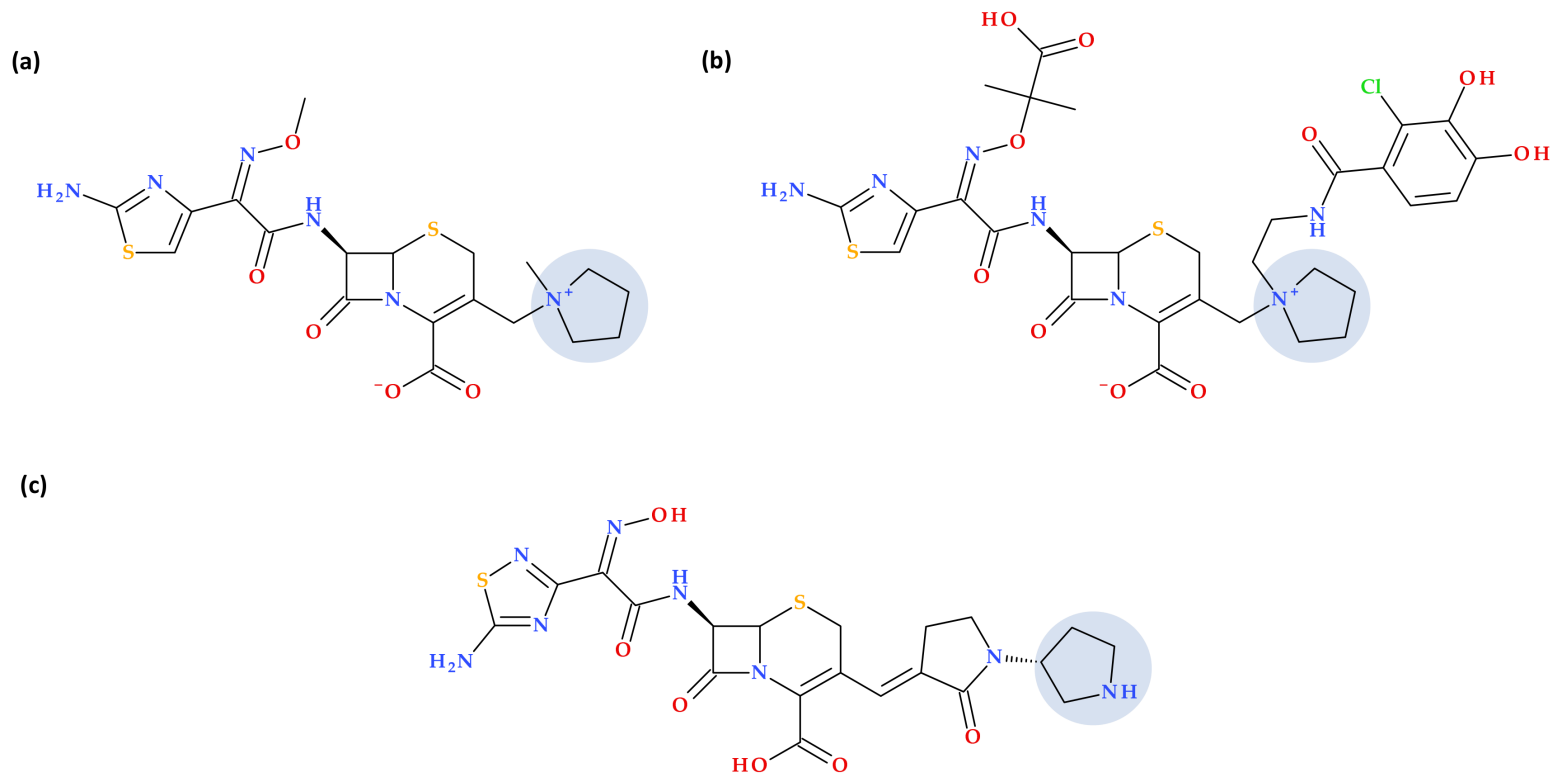
Molecular structure of (**a**) Cefepime, (**b**) Cefiderocol, highlighting the pyrrolidinium group; and (**c**) Ceftobiprole.

*Cefepime* is a fourth-generation cephalosporin approved by the FDA in 1996 and widely used for the treatment of severe nosocomial and community-acquired infections, including pneumonia, febrile neutropenia, UTIs, intra-abdominal infections, and infections caused by multidrug-resistant (MDR) Gram-negative pathogens. Due to its zwitterionic structure (Figure 3a), cefepime exhibits rapid outer membrane permeation in Gram-negative bacteria. It demonstrates enhanced stability against many chromosomal and plasmid-mediated beta-lactamases compared with earlier cephalosporins [68].

Cefepime contains an *N*-methylpyrrolidinium moiety at the C-3 position of the cephalosporin nucleus. The quaternary pyrrolidine ring is a key optimisation that confers several advantageous pharmacological properties. The positively charged pyrrolidinium group contributes to the molecule’s zwitterionic character, facilitating passage through bacterial porin channels and increasing accumulation within Gram-negative organisms. Consequently, cefepime displays potent activity against members of the *Enterobacterales* family, *Pseudomonas aeruginosa*, and numerous other clinically relevant pathogens [68,69].

In addition to improving bacterial cell penetration, the pyrrolidinium substituent contributes to cefepime’s relative resistance to hydrolysis by many beta-lactamases. It enhances its affinity for multiple penicillin-binding proteins (PBPs), thereby strengthening inhibition of bacterial cell-wall synthesis. Because of this favourable combination of broad-spectrum activity, beta-lactamase stability, and pharmacokinetic properties, Cefepime remains an important therapeutic option for the management of serious Gram-negative infections. It is frequently used as a component of newer beta-lactam/beta-lactamase inhibitor combinations, such as Cefepime/Enmetazobactam [68]. This example illustrates how introducing a positively charged pyrrolidinium group can simultaneously enhance membrane permeability, target accessibility, and beta-lactamase stability without compromising antibacterial potency.

*Cefiderocol* is a new siderophore cephalosporin with promising in vitro activity against Gram-negative bacilli, developed by Shionogi in Osaka, Japan. In 2019, Cefiderocol was approved by the FDA for the treatment of complicated UTIs and pyelonephritis. Also, in 2020, the EMA approved its use for infections caused by aerobic Gram-negative bacteria, hospital-acquired pneumonia, and ventilator-associated bacterial pneumonia [70]. At the C-3 side chain, Cefiderocol has a pyrrolidinium group (Figure 3b), which increases potency against drug-resistant Gram-negative bacteria by conferring stability to beta-lactamases while maintaining affinity to the molecular target (the PBP) and offers amphoteric character due to a quaternary nitrogen atom that increases the water solubility of the molecule [56,71,72]. The pyrrolidinium substituent complements this siderophore strategy by preserving favourable physicochemical properties after active transport into the bacterial periplasm. Cefiderocol is an interesting new antibiotic that acts like a “Trojan horse,” entering the bacterial cell’s outer membrane and the periplasm via specific iron transporter channels. Also, Cefiderocol has shown stability against hydrolysis by serine and metallo-lactamases, including those associated with significant carbapenemases [73,74].

*Ceftobiprole* is a 5th-generation cephalosporin for parenteral use, with bactericidal activity against both MRSA and methicillin-susceptible *Staphylococcus aureus* (MSSA). The antibiotic was approved in 2024 as Ceftobiprole medocaril (Zevtera) and is indicated for the treatment of *Staphylococcus aureus* bloodstream infections (bacteremia) (SAB), including those with right-sided infective endocarditis, acute bacterial skin and skin structure infections (ABSSSI), and community-acquired bacterial pneumonia (CABP) [75,76]. Ceftobiprole is considered a pyrrolidinone-3-ylidene-methyl cephalosporin with extended activity against Gram-negative pathogens, such as *Pseudomonas aeruginosa* and *Enterobacteriaceae*, and against Gram-positive pathogens, including MRSA, penicillin-resistant *Streptococcus pneumoniae*, and *Enterococcus faecalis* [77,78].

MRSA’s high resistance to beta-lactam antibiotics is known to be facilitated by the expression of PBP2a [79]. The planar pyrrolidine ring in the structure of this antibiotic makes the compound resemble the rest of the pentaglycine (Figure 3c). According to a molecular docking study by Morosini M.-I. et al. (2019) [80], Ceftobiprole binds to the active sites of PBP2a and 2x, resulting in a complex with the acyl-antibiotic PBP2a. Within the complex, the pyrrolidine ring lies between the tyrosine (Tyr446), the methionine (Met641), and the threonine (Thr600) residues. The pyrrolidine forms a hydrogen bond with the sulfur atom from the methionine residue (Met641). Simultaneously, this heterocycle forms a π-π interaction with the tyrosine residue (Tyr446) and hydrophobic interactions with the threonine residues (Thr600) [80,81].

#### 2.2.2. Fluoroquinolones

Fluoroquinolones are a class of bactericidal antimicrobial agents, whose clinical efficacy is based on selective inhibition of enzymes essential for bacterial DNA topology and replication: DNA gyrase and topoisomerase IV. The basic pharmacophore skeleton, 1,4-dihydro-4-oxoquinoline, has undergone continuous rational optimisation, with substitution at the C-7 position proving critical for modulating pharmacokinetic properties and the spectrum of activity. A major qualitative stage in the development of this class is the transition from classical derivatives based on the piperazine ring to the integration of the pyrrolidine nucleus [57,82]. Pyrrolidine was included in the structures of these compounds to enhance their activity against Gram-positive bacteria, including MRSA, and to improve their pharmacokinetic profile (increased half-life) and bioavailability [83,84].

**Figure 4:**
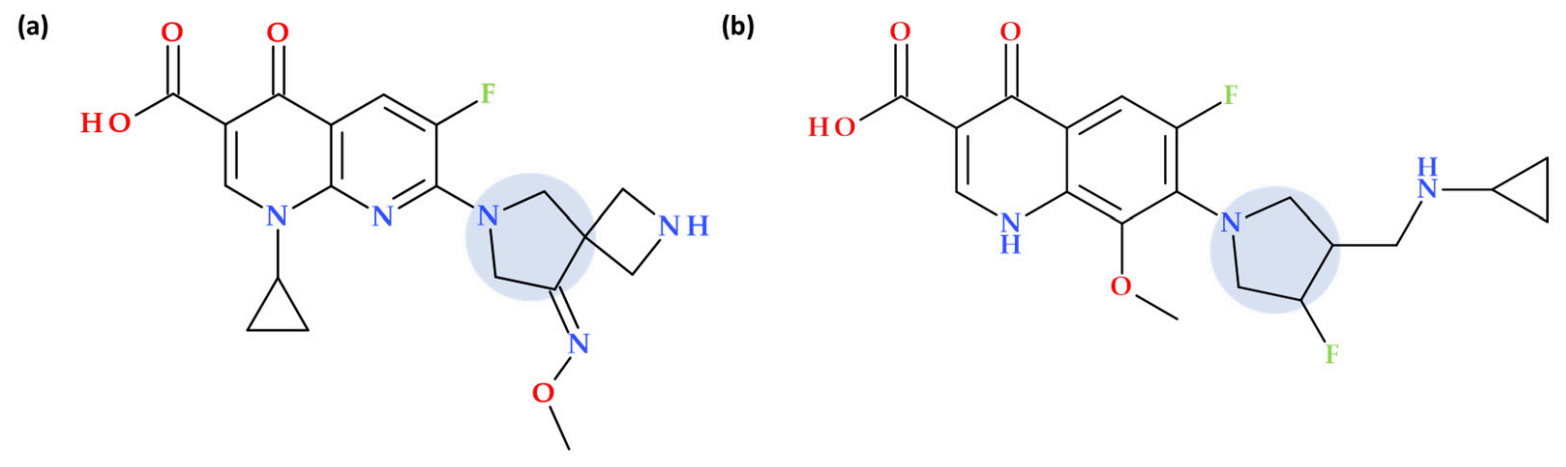
The molecular structure of (**a**) Zabofloxacin and (**b**) Lascufloxacin.

*Zabofloxacin* was developed by Dong Wha Pharm. Co., Ltd. Seoul, South Korea and was approved in South Korea in 2015; it is used for the treatment of acute bacterial exacerbation of chronic obstructive pulmonary disease via oral administration [85,86]. The spectrum of activity of Zabofloxacin includes Gram-negative and Gram-positive respiratory pathogens, especially *Streptococcus pneumoniae* and drug-resistant *Neisseria gonorrhoeae* [87]. At the C-7 position, Zabofloxacin has an unusual bicycle formed by fusion of azetidine with pyrrolidine (2,6-diazaspiro [3.4]octane) to which an 8-methoxyimino group is added (Figure 4a). Zabofloxacin is well tolerated in clinical practice [88].

*Lascufloxacin* was developed by Kyorin Pharmaceuticals Co., Ltd. in Japan, and was approved in 2019 (Japan) for the treatment of respiratory infections, including community-acquired pneumonia and ear, nose, throat infections [89,90,91]. At the C-7 position, Lascufloxacin has an interesting heterocyclic group: a main pyrrolidine heterocycle substituted with a (cyclopropylamino)methyl moiety (Figure 4b). The pyrrolidine nucleus enhances activity against Gram-positive bacteria; therefore, it has great potential for treating respiratory and ear, nose, and throat infections [92].

The two fluoroquinolone examples demonstrate that substituting the classical C-7 piperazine ring with pyrrolidine-containing moieties is an effective strategy for expanding Gram-positive activity while improving pharmacokinetic performance.

#### 2.2.3. Lincosamides

**Figure 5:**
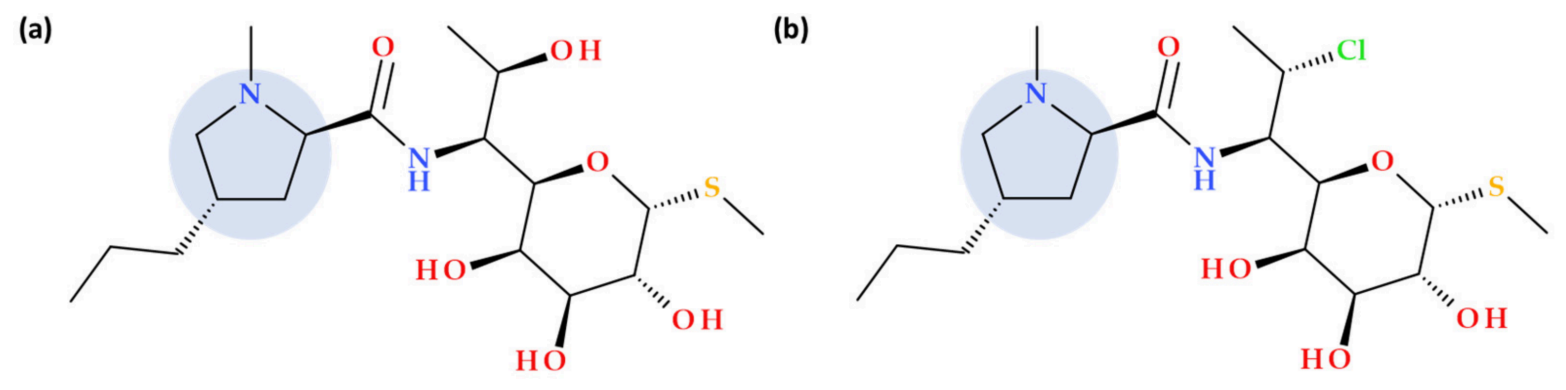
The molecular structure of (**a**) Lincomycin and (**b**) Clindamycin.

Lincosamides are a class of antibiotics represented primarily by lincomycin and its semisynthetic derivative Clindamycin (Figure 5). Structurally, these compounds contain a propylproline-derived pyrrolidine ring linked through an amide bond to a methylthio-lincosamide sugar moiety. The pyrrolidine scaffold is a structural key element that contributes to the conformation necessary for interaction with the bacterial ribosome [93,94].

Lincosamides exert their antibacterial activity by binding to the 23S rRNA of the 50S ribosomal subunit, thereby inhibiting bacterial protein synthesis. They are particularly effective against Gram-positive bacteria and anaerobic pathogens. Among this class, Clindamycin is the most widely used clinically due to chlorination of lincomycin, which enhances antibacterial potency, oral absorption, and tissue penetration [93,94,95].

*Clindamycin* is the most clinically significant lincosamide antibiotic and a prominent example of a pyrrolidine-derived antibacterial agent. Structurally, it consists of a substituted pyrrolidine (propylproline) ring linked via an amide bond to a methylthio-lincosamide sugar. The pyrrolidine moiety is essential for maintaining the three-dimensional conformation required for high-affinity binding to the biological target, thereby inhibiting protein synthesis [93,95].

Clindamycin is a semisynthetic derivative of Lincomycin in which the 7(*R*)-hydroxyl group is replaced by chlorine (Figure 5b). The chlorine substituent significantly improves lipophilicity, oral bioavailability, tissue penetration, and antibacterial potency compared with the parent compound. Nowadays, Clindamycin is widely used to treat infections caused by Gram-positive cocci, including *Staphylococcus aureus* and *Streptococcus* spp., as well as various anaerobic bacteria [93]. The pyrrolidine ring contributes not only to the molecular stability of the drug, but also to the key interactions responsible for its antibacterial effect [95,96].

#### 2.2.4. Streptogramins

Streptogramins are a distinctive class of protein-synthesis inhibitors originally isolated from *Streptomyces pristinaespiralis*. Chemically, the streptogramins comprise two structurally distinct groups, streptogramin A and streptogramin B, which act synergistically. Individually, each component generally exhibits bacteriostatic activity; however, when administered together, they produce a rapid bactericidal effect against many Gram-positive pathogens, including MDR strains [94,97,98].

**Figure 6:**
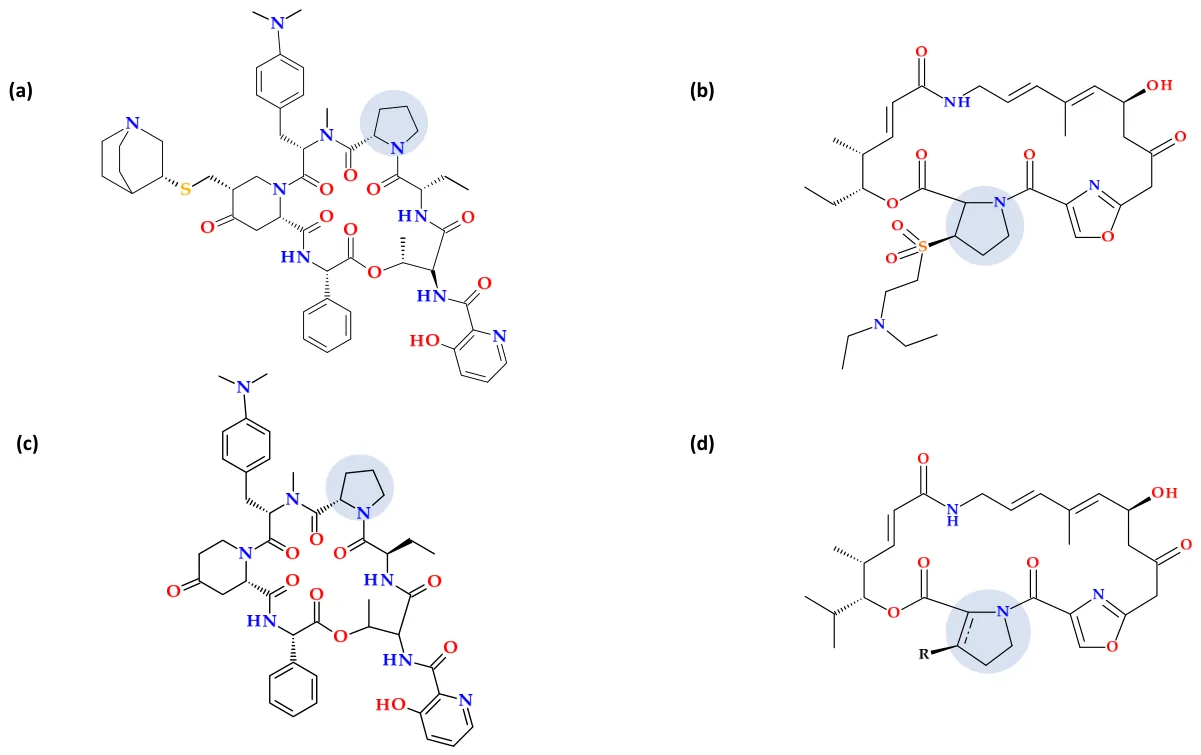
Molecular structure of (**a**) Quinupristin, (**b**) Dalfopristin, (**c**) Pristinamycin IA, and (**d**) Pristinamycin IIA/IIB (IIA: R = H, Δ-26-27)/IIB: R = H, 27-R), highlighting the pyrrolidine-containing moiety [100,101].

The clinically relevant streptogramin combination is Quinupristin/Dalfopristin (Synercid^®^), a semisynthetic derivative of Pristinamycin composed of Quinupristin (Streptogramin B) and Dalfopristin (Streptogramin A) in a 30:70 ratio. Structurally, Dalfopristin is derived from Pristinamycin IIA, a polyunsaturated cyclic macrolactone. In contrast, Quinupristin originates from Pristinamycin IA, a cyclic hexadepsipeptide containing a substituted pyrrolidine-derived amino acid residue within its macrocyclic structure (Figure 6). The presence of a pyrrolidine-containing fragment contributes to the conformational rigidity required for high-affinity ribosomal binding [60,94,99,100,101].

In this series, antibacterial activity was influenced by the substitution patterns of the benzyl and carboxamide regions rather than solely by the pyrrolidine nucleus. Although several derivatives inhibited both Gram-positive and Gram-negative strains, the available inhibition-zone data should be regarded as preliminary, as MIC, cytotoxicity, and mechanistic studies were not reported.

Streptogramins inhibit bacterial protein synthesis through sequential binding to the 50S ribosomal subunit. Dalfopristin initially binds to the peptidyl-transferase centre of the 23S rRNA, inducing a conformational change that markedly increases the affinity of the ribosome for Quinupristin. Subsequently, Quinupristin binds within the ribosomal exit tunnel, blocking peptide elongation and promoting the release of incomplete peptide chains. The synergistic interaction between both compounds results in irreversible inhibition of protein synthesis and explains their bactericidal activity when used in combination [94,97,99].

*Quinupristin/Dalfopristin* is active primarily against Gram-positive bacteria, including MRSA, coagulase-negative staphylococci, penicillin-resistant *Streptococcus pneumoniae*, and vancomycin-resistant *Enterococcus faecium* (VRE). The combination was approved for the treatment of complicated skin and skin-structure infections and severe infections caused by susceptible VRE isolates. However, clinical use remains limited due to adverse effects such as arthralgia, myalgia, infusion-related reactions, and significant CYP3A4-mediated drug interactions [60,98].

Streptogramins provide an interesting example of how pyrrolidine-containing structural motifs contribute to antibacterial activity. The pyrrolidine moiety enhances molecular conformation and facilitates interactions with the ribosomal target, contributing to the remarkable synergistic antibacterial activity of this class. The successful clinical development of Quinupristin/Salfopristin demonstrates the continued therapeutic value of pyrrolidine-containing scaffolds in the treatment of infections caused by MDR Gram-positive pathogens [98].

*Pristinamycin* is a valuable antibiotic with limited uses. It has been extensively used for more than 50 years in France and parts of Africa as a therapeutic option for Gram-positive bacterial infections, particularly those associated with multidrug resistance [98,102]. Pristinamycin IIA is a polyunsaturated cyclic macrolactone, and Pristinamycin IA is a cyclic hexa-depsipeptide (Figure 6). The two streptogramins are bacteriostatic when used separately, but they become bactericidal when combined. Both compounds bind different targets in the peptidyltransferase domain of the 50S ribosomal subunit. Pristinamycin is used orally and topically for cutaneous, respiratory, and bone infections caused by staphylococci [103,104]. Semisynthetic modification of Pristinamycin IA and Pristinamycin IIB (Figure 6) to improve aqueous solubility enabled the development of the parenteral Quinupristin/Dalfopristin combination, a streptogramin antibacterial agent used against MDR pathogens such as VRE and vancomycin-resistant *Staphylococcus aureus* (VRSA) [100].

#### 2.2.5. Tetracyclines

**Figure 7:**
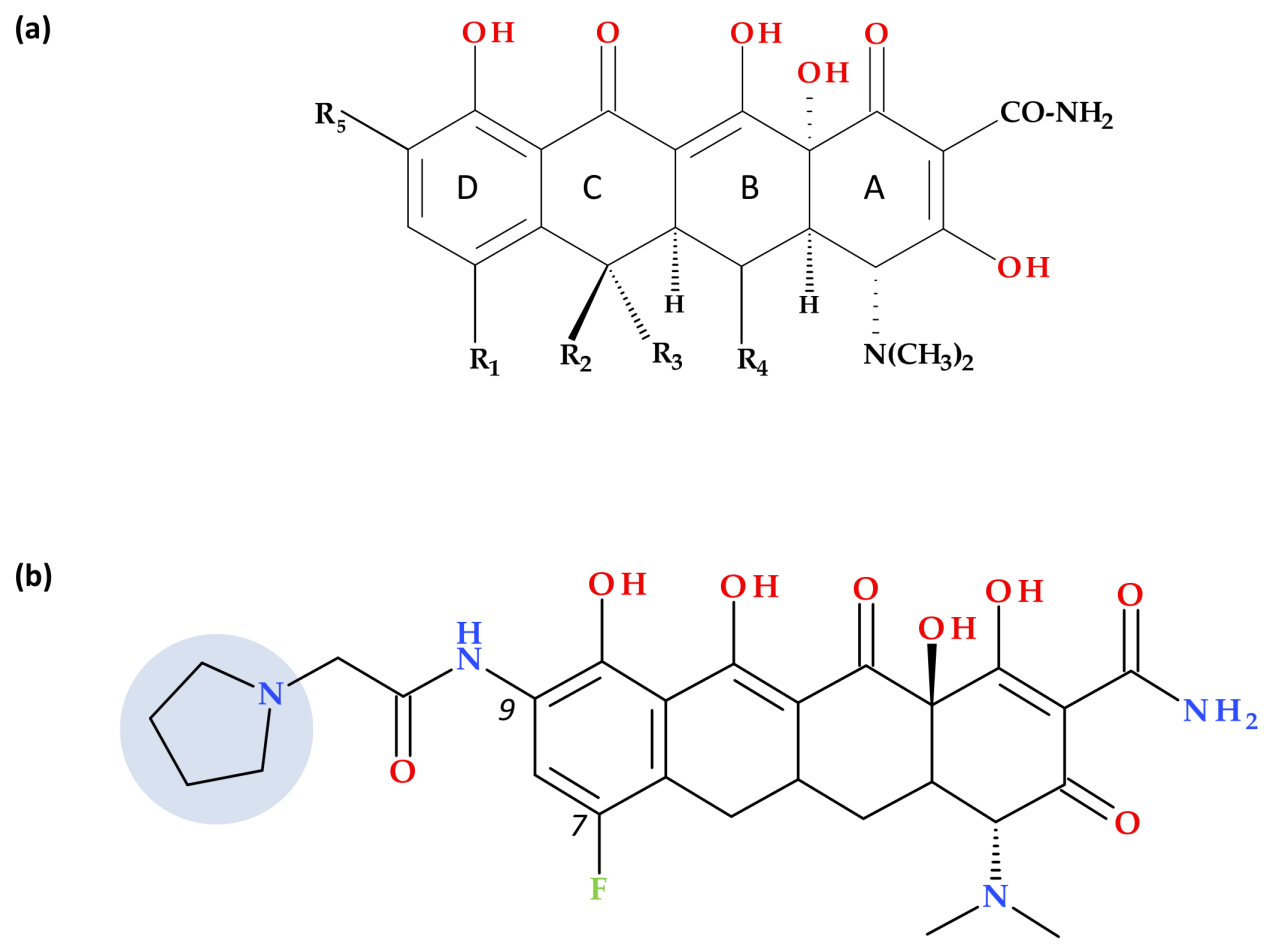
(**a**) General molecular structure of tetracyclines, and (**b**) Eravacycline molecular structure, highlighting the pyrrolidine-containing moiety.

Tetracyclines are a classic class of broad-spectrum antibiotics whose bacteriostatic activity is based on reversible inhibition of protein synthesis by binding to the 30S ribosomal subunit, thereby blocking the attachment of aminoacyl-tRNA [105]. A fused linear tetracyclic system has been effective for decades; the clinical utility of older generations has been severely compromised by the proliferation of resistance mechanisms, particularly efflux pumps and ribosomal protective proteins. Recent efforts in medicinal chemistry have focused on structural modifications of the D-ring (Figure 7a). A remarkable innovation in the development of next-generation molecules (such as fluorocyclines) is the integration of the pyrrolidine nucleus into their classic skeleton [106,107].

*Eravacycline* is a novel, unique synthetic fluorocycline antibacterial agent with broad-spectrum activity, consisting of a tetracyclic core scaffold with unique modifications in the D ring of the naphtacen nucleus, a pyrrolidinoacetamido group at the C-9 position (Figure 7b), which is a highly polar moiety. It is considered a modified tetracycline, developed to overcome common mechanisms of tetracycline resistance, such as efflux pumps, ribosomal protection, and enzymatic inactivation [108,109,110,111]. Eravacycline exhibits potent activity against a broad spectrum of clinically relevant Gram-positive and Gram-negative bacteria, including aerobic and anaerobic organisms as well as MDR strains.

The introduction of the pyrrolidine-containing side chain at the C-9 position constitutes a major structural innovation in Eravacycline, conferring enhanced target affinity and significantly improving activity against tetracycline-resistant pathogens [112]. The pyrrolidine moiety creates a steric barrier that diminishes efflux pump recognition and transport, thereby promoting intracellular retention of the antibiotic. Simultaneously, this structural feature increases the molecule’s affinity for its ribosomal target, leading to more effective inhibition of protein synthesis [108,113,114]. Eravacycline is a unique tetracycline due to the combination of the fluorine atom at position C-7 and the pyrrolidine ring at position C-9, which confer an optimised pharmacokinetic profile owing to its lipophilicity and electronic reactivity. These two changes offer the molecule exceptional activity against MDR pathogens, including carbapenemase-producing *Enterobacteriaceae* (CRE) [112,115].

The successful development of Eravacycline illustrates how rational incorporation of a pyrrolidine moiety can revitalise an established antibiotic class by enhancing efficacy against MDR pathogens.

## 3. Pyrrolidine-Based Antibacterial Candidates: Natural Products, Derivatives, and Synthetic Analogues

This section is dedicated specifically to pyrrolidine-containing compounds that have been investigated as antibacterial candidates. The compounds are discussed according to their chemical nature, pharmacological relevance, and medicinal chemistry characteristics, with emphasis on antibacterial activity, structure-activity relationships, and potential therapeutic applications.

Various pyrrolidine derivatives are currently being explored as drug candidates, including those created and researched for their potential as antibacterial agents [42]. Pyrrolidines, prevalent in many natural products, form the backbone of the amino acid proline, which has led to increased interest in this heterocycle. These derivatives are effectively used in ligand design due to their chirality in transition-metal- and organocatalytic reactions. Substituted pyrrolidine derivatives have a variety of bioactivities, including antibiotic, antibacterial, antifungal, and cytotoxic actions. As a result, they offer a great opportunity to develop new drugs [116,117].

In addition, the diastereoselective and enantioselective synthesis of stereochemical models of various substituted pyrrolidines has advanced significantly, and it is now possible to create two distinct stereoisomers of pyrrolidine from the same reaction partners with increased bioactivity [21]. The sp3-hybridisation’s ability to efficiently explore the pharmacophore space, the contribution to the molecule’s stereochemistry, and the increased three-dimensional (3D) coverage caused by the ring’s non-planarity (“pseudorotation”) all contribute to the pyrrolidine heterocycle’s high level of interest [16].

### 3.1. 1-Acetyl-2-Benzylpyrrolidine-2-Carboxylic Acid Derivatives

**Figure 8:**
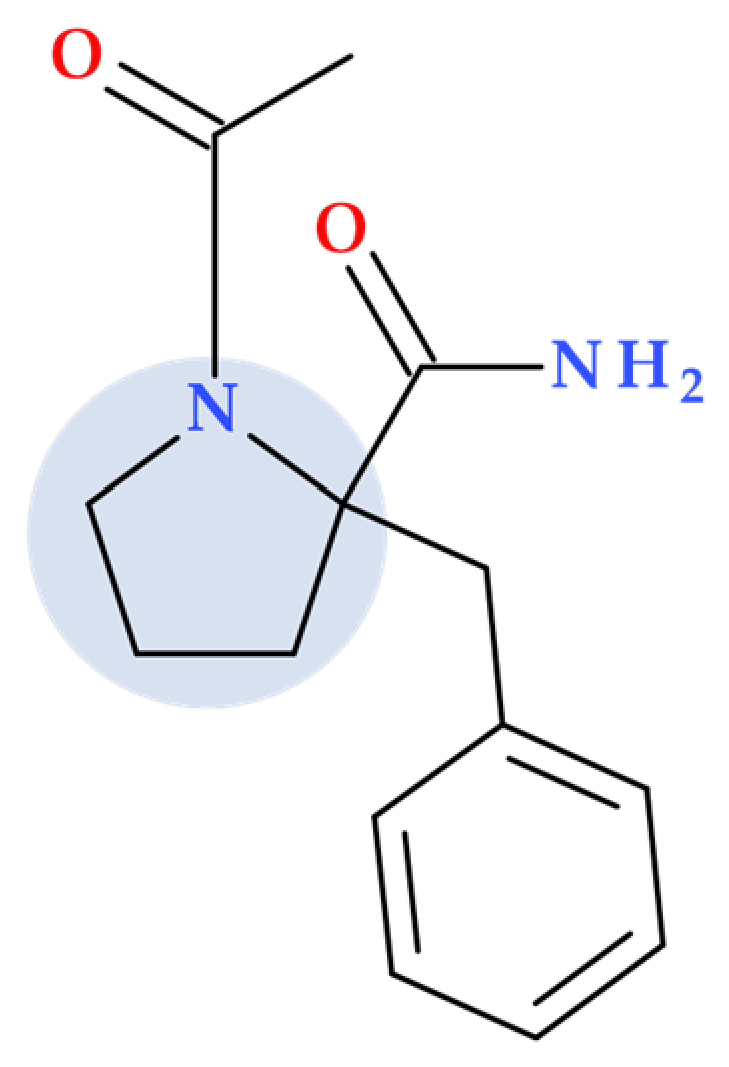
Molecular structure of the compound 1-acetyl-2-benzylpyrrolidine-2-carboxamide, highlighting the pyrrolidine-containing moiety [118].

According to Sreekanth and Jha (2020), a series of 1-acetyl-2-benzylpyrrolidine-2-carboxylic acid derivatives was synthesised and evaluated for antibacterial activity against both Gram-positive and Gram-negative bacteria, with Chloramphenicol as the reference drug [118]. Several derivatives exhibited promising antibacterial effects against *Escherichia coli*, *Pseudomonas aeruginosa*, *Bacillus subtilis*, and *Staphylococcus aureus*. Compound 1-acetyl-2-benzylpyrrolidine-2-carboxamide (Figure 8) was the most active derivative in the series, exhibiting MIC values of 32 and 64 μg/mL against *Escherichia coli* and *Pseudomonas aeruginosa*, respectively, and enhanced activity against Gram-positive bacteria (*Staphylococcus aureus* and *Bacillus subtilis*; MIC = 16 μg/mL). The obtained results highlighted the potential of the pyrrolidine scaffold for the development of new antibacterial agents [118].

### 3.2. Alkaloids and Derivatives

#### 3.2.1. Scalusamides A, B, and C

**Figure 9:**
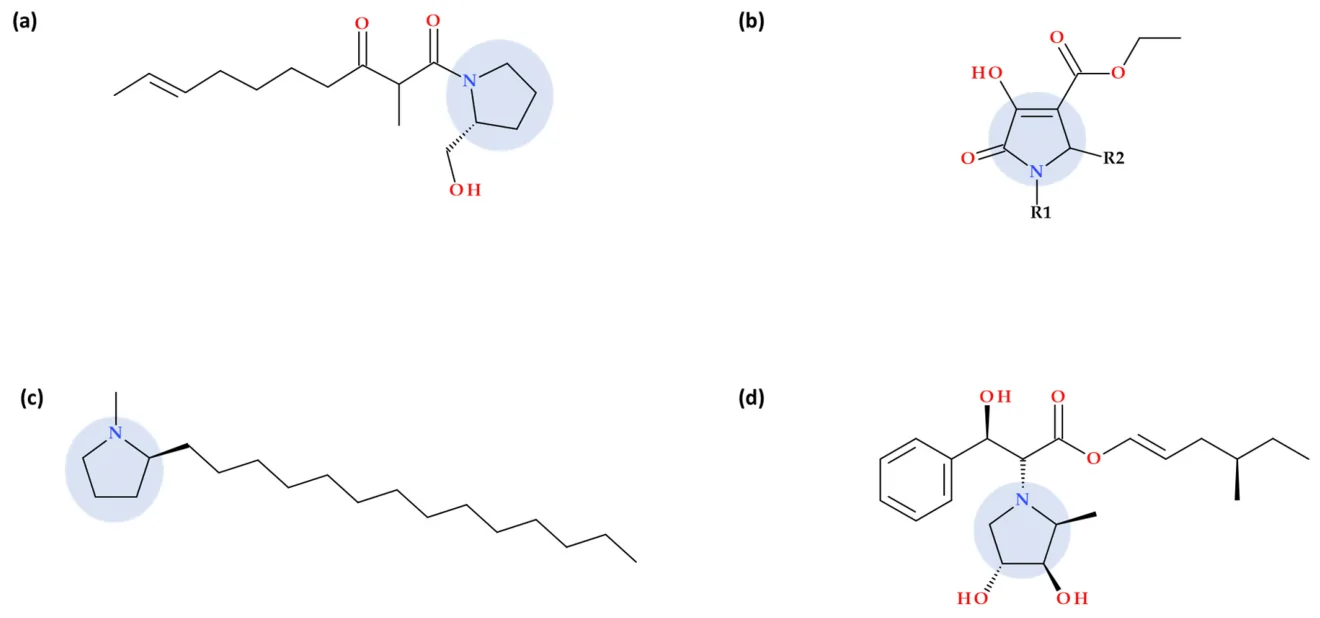
Molecular structure of (**a**) Scalusamide A, (**b**) MFM 501, (**c**) (*R*)-Bgugaine, and (**d**) Vibripyrrolidine.

Three pyrrolidine alkaloids, Scalusamides A, B, and C, were found in the cultured broth of the fungus *Penicillium citrinum*, isolated from the gastrointestinal tract of a marine fish. *Penicillium brevicompactum*, *Cryptococcus neoformans*, and *Micrococcus luteus* were all susceptible to the antifungal and antibacterial effects of Scalusamide A (Figure 9a) [49]. The pyrrolidine nucleus is the central pharmacophoric element of scalusamides A–C and represents a valuable heterocyclic scaffold for this family of marine alkaloids. Structural characterisation revealed the presence of C-7 epimers, and biological evaluation showed that scalusamide A possesses antibacterial and antifungal activity (MIC = 33 μg/mL) [49,50].

#### 3.2.2. MFM 501

As analogues for natural polyhydroxy alkaloids, 28 novel pyrrolidine-type compounds and new Codonopsinine derivatives were discovered. Their antibacterial efficacy was tested against a panel of *Staphylococcus aureus* strains, demonstrating good inhibitory activity and effectiveness against various isolates [45].

MFM 501, a pyrrolidine derivative (Figure 9b), demonstrated notable antibacterial activity against a panel of 55 *Staphylococcus aureus* clinical isolates (43 MRSA and 12 MSSA), with MIC values ranging from 15.6 to 31.3 μg/mL. In contrast, the compound exhibited Minimum Bactericidal Concentration (MBC) values of 250–500 μg/mL against 58 *Staphylococcus aureus* isolates (45 MRSA and 13 MSSA). The substantial difference between MIC and MBC values indicates that MFM 501 exerts a predominantly bacteriostatic rather than bactericidal effect against both MRSA and MSSA strains [45]. The antibacterial activity of MFM 501 supports the importance of the pyrrolidine scaffold in the design of novel anti-staphylococcal agents.

#### 3.2.3. (*R*)-Norbgugaine

(*R*)-Bgugaine is a natural pyrrolidine alkaloid isolated from *Arisarum vulgare*, while (*R*)-Norbgugaine is its synthetic demethylated analogue (Figure 9c). Biological studies demonstrated that (*R*)-Norbgugaine possesses pronounced anti-virulence activity against *Pseudomonas aeruginosa* by inhibiting several quorum-sensing-regulated pathogenicity factors, including swarming motility, biofilm formation, pyocyanin production, rhamnolipid synthesis, and LasA protease activity, highlighting the potential of pyrrolidine alkaloids as anti-infective agents [119]. Biological evaluation demonstrated that (*R*)-Norbgugaine inhibits *Pseudomonas aeruginosa* biofilm formation in a concentration-dependent manner, with a maximum inhibition of 83% at 1.8 mM or higher. Importantly, no effect on bacterial growth was detected, supporting its role as a quorum-sensing and anti-virulence agent rather than a direct antibacterial compound [119]. MIC values have not yet been reported. The potent anti-biofilm and quorum-sensing inhibitory properties of (*R*)-Norbgugaine highlight the significance of the pyrrolidine nucleus in anti-infective drug design.

#### 3.2.4. Vibripyrrolidine

Vibripyrrolidine is a pyrrolidine alkaloid (Figure 9d) isolated from the marine bacterium *Vibrio ruber*. Marine microorganisms are recognised as a rich source of structurally unique natural products with diverse biological activities. Biological evaluation demonstrated that Vibripyrrolidine possesses notable antibacterial activity against *Staphylococcus aureus*, with an MIC value of 1.95 μg/mL [120]. The potent activity observed for this natural product demonstrates the relevance of the pyrrolidine ring in the design of new antibacterial compounds.

### 3.3. Anisomycin

**Figure 10:**
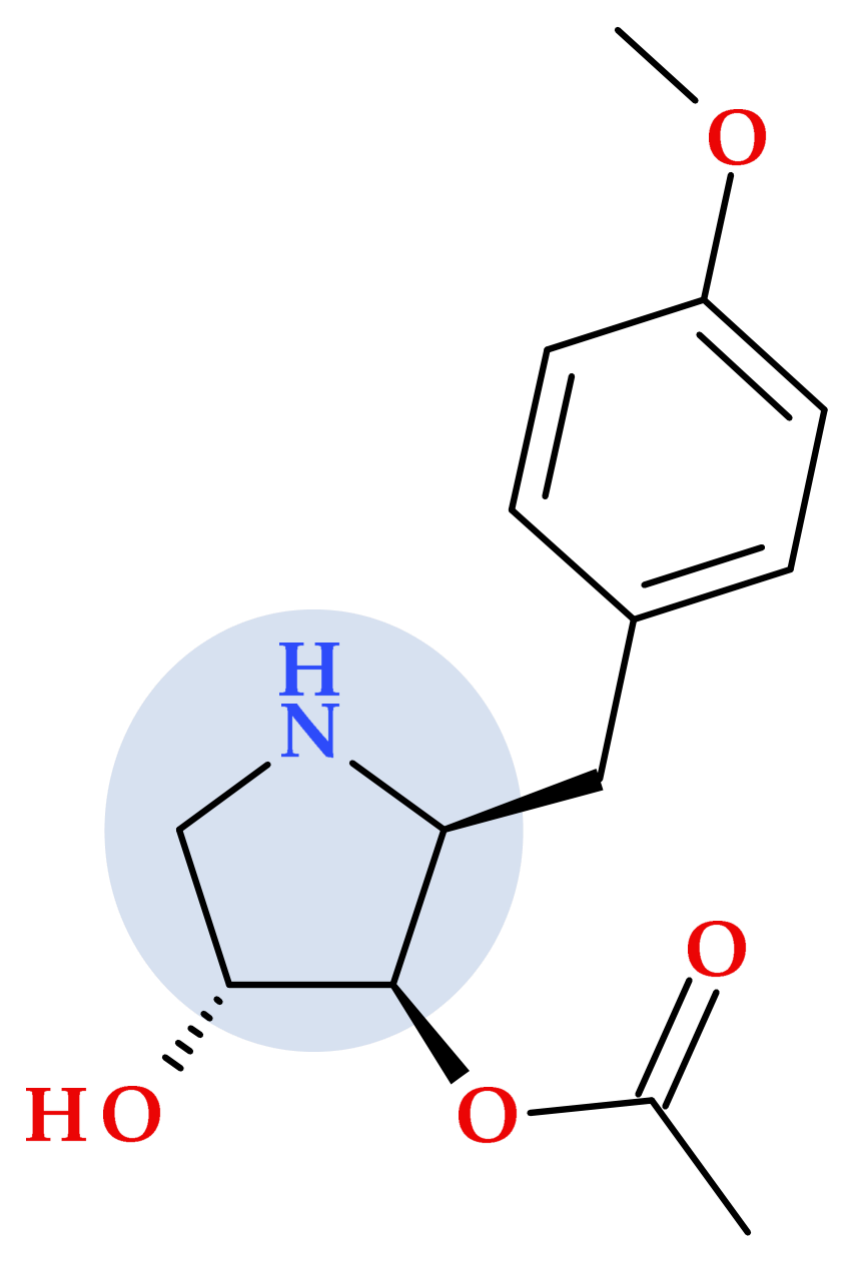
The molecular structure of Anisomycin.

Anisomycin (Flagecidin) is a naturally occurring monohydroxypyrrolidine antibiotic (Figure 10) isolated from *Streptomyces griseolus*. The compound inhibits protein and DNA synthesis by targeting the peptidyl transferase activity of the 60S subunit of eukaryotic 80S ribosomes, thereby blocking peptide bond formation, arresting translational elongation, and stabilising polyribosomes [121]. Anisomycin has multiple roles, including as an antibacterial, antiviral, antiparasitic, and antineoplastic agent. Also, Anisomycin may have clinical significance, as it exhibits numerous biological and pharmacological properties [121,122]. In addition, Anisomycin demonstrated potent in vitro antiviral activity against Group B Coxsackievirus type 3 (CVB3), markedly suppressing viral replication at submicromolar concentrations with minimal cytotoxicity [123]. Thus, Anisomycin is a valuable pyrrolidine-based natural scaffold for the development of novel anti-infective and therapeutic agents.

### 3.4. N-Benzoylthiourea-Pyrrolidine Derivatives

**Figure 11:**
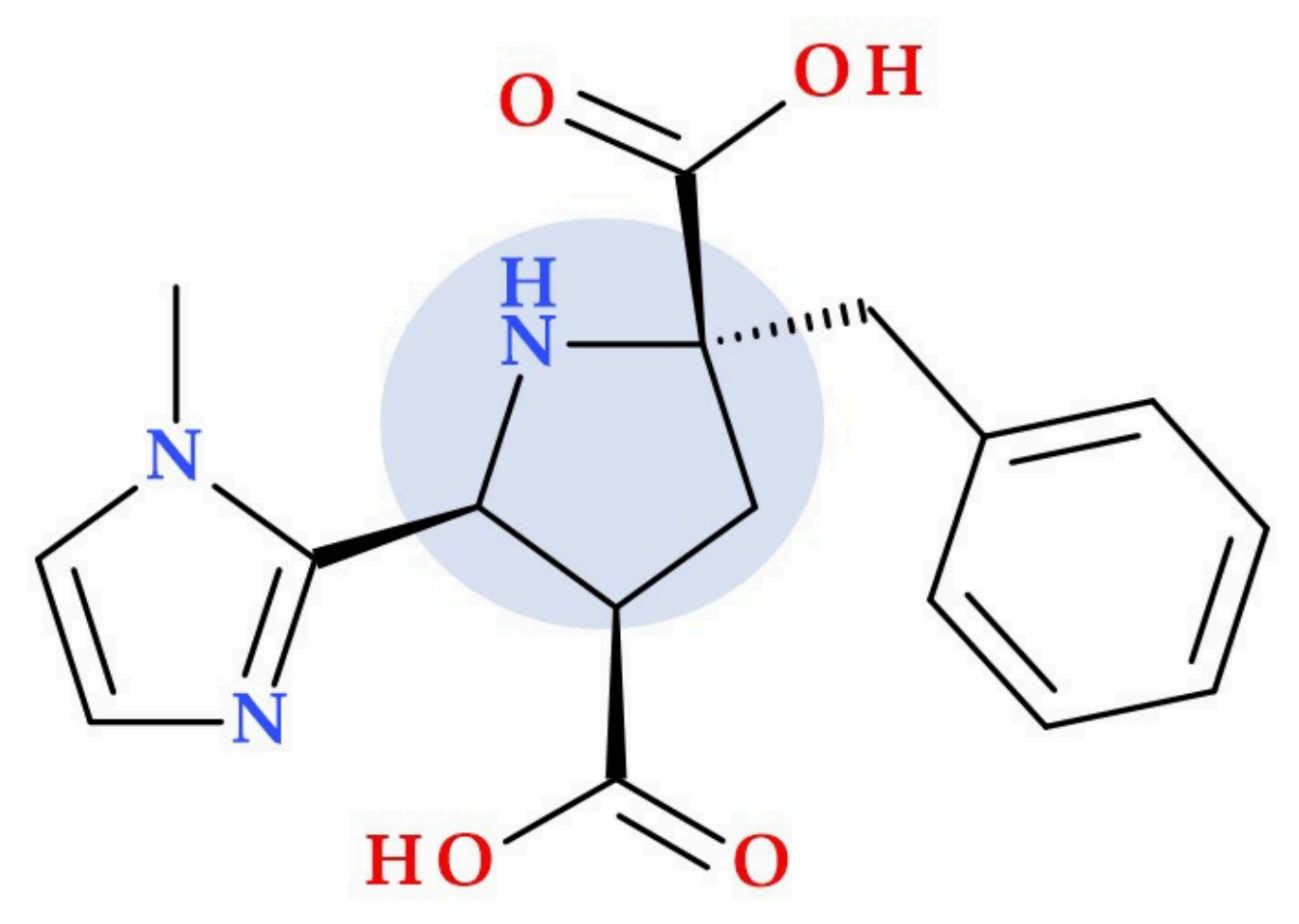
The molecular structure of the compound (2*RS*,4*SR*,5*RS*)-2-benzyl-5-(1-methyl-1*H*-imidazol-2-yl)pyrrolidine-2,4-dicarboxylate [124].

A series of *N*-benzoylthiourea-pyrrolidine derivatives containing an imidazole moiety was synthesised and evaluated for biological activity. Two derivatives showed moderate antibacterial activity, particularly against *Acinetobacter baumannii* (MIC = 62.5 μg/mL). In addition, the newly synthesised pyrrolidine derivatives exhibited MIC values ranging from 15.62 to 31.25 μg/mL against the *Mycobacterium tuberculosis* H37Rv strain. A single compound, dimethyl (2*RS*,4*SR*,5*RS*)-2-benzyl-5-(1-methyl-1*H*-imidazol-2-yl)pyrrolidine-2,4-dicarboxylate (“1bb”) (Figure 11), with a MIC value of 15.62 μg/mL, was the most effective against this *Mycobacterium tuberculosis* H37Rv strain [124]. The observed antibacterial and antitubercular activities highlight the pyrrolidine as a scaffold in anti-infective drug design. In combination with the benzoylthiourea and imidazole pharmacophores, the pyrrolidine likely facilitates enhanced target binding and contributes to the overall antimicrobial profile of these derivatives.

### 3.5. Carbapenems

#### 3.5.1. Benapenem

**Figure 12:**
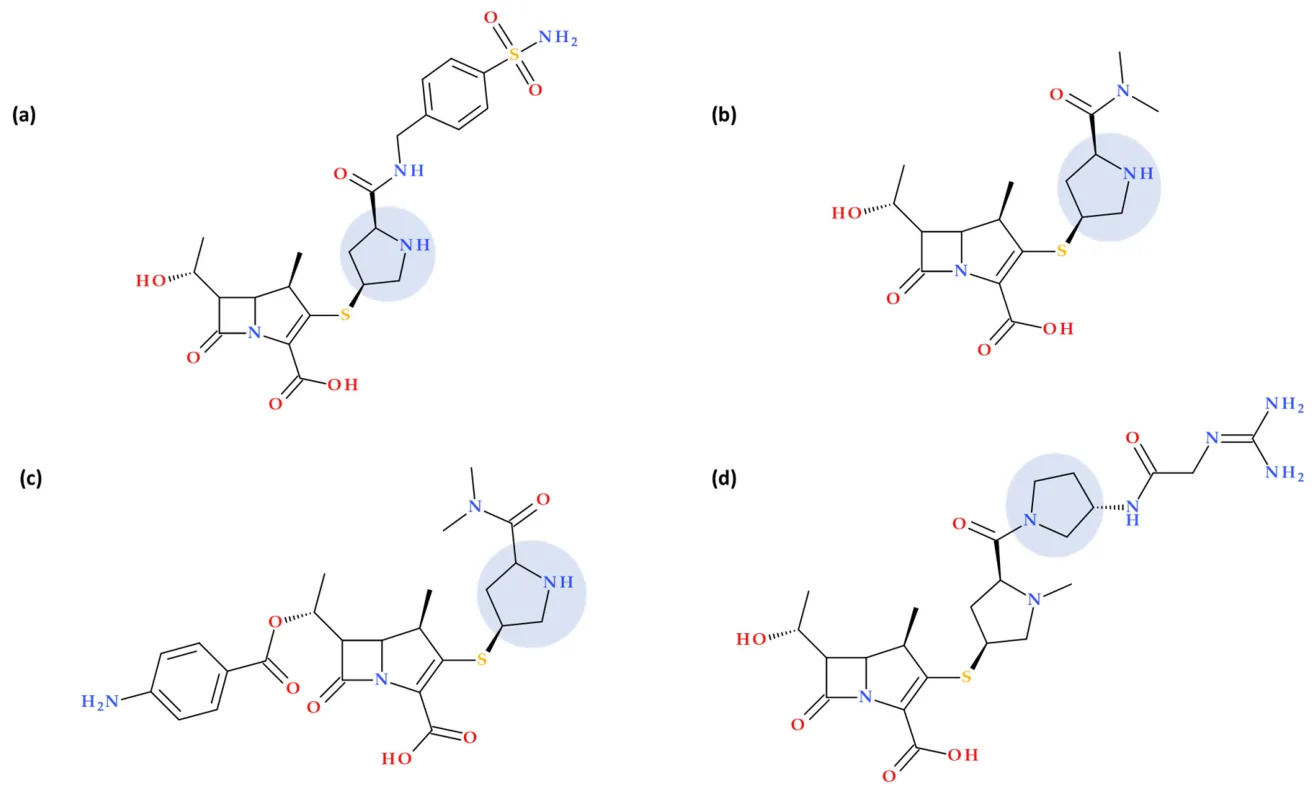
The molecular structure of (**a**) Benapenem, (**b**) Meropenem, (**c**) 6-[(1-(4-aminobenzoyloxy)ethyl)]-3-[(5-(dimethylcarbamoyl)pyrrolidin-3-yl)thio]-4-methyl-7-oxo-1-azabicyclo[3.2.0]hept-2-ene-2-carboxylic acid, and (**d**) Tomopenem.

Benapenem is a novel carbapenem β-lactam antibiotic developed by Sihuan Pharmaceutical (Beijing, China), which completed Phase II/III clinical trials in 2020 as an intravenous therapy for complicated UTI, including pyelonephritis [125,126,127]. Structurally, Benapenem is similar to Ertapenem and has a comparable extended half-life of 7 hours. As a result, it facilitates once-daily intravenous administration, identical to that of Ertapenem, which gives it a competitive advantage over other carbapenems, whose shorter half-lives require multiple daily doses [127]. A methyl-benzyl sulfonamide moiety was added to the carbapenems’ traditional structure to improve molecular weight and lipophilicity. Additionally, like Ertapenem, Benapenem is a pyrrolidine derivative (Figure 12a) [128]. Benapenem has been shown to have excellent antibacterial activity against various bacteria, including Gram-negative and anaerobic bacteria, as well as extended-spectrum beta-lactamase (ESBL)-positive bacteria. The antibiotic exhibited potent in vitro activity against *Enterobacteriaceae*, with MIC90 values ≤ 0.5 μg/mL for most tested species, including ESBL-producing *Escherichia coli* and *Klebsiella pneumoniae*. Reduced susceptibility was observed for *Providencia* spp. (MIC90 = 4 μg/mL) and *Enterobacter cloacae* (MIC90 = 1 μg/mL) [129]. The molecular structure of Benapenem demonstrates that incorporating a pyrrolidine moiety can be successfully used to design potent, broad-spectrum antibacterial drugs.

#### 3.5.2. Meropenem Ester Derivatives

To create structural analogues of a beta-lactam antibiotic effective against resistant bacterial strains, ester derivatives of Meropenem (Figure 12b) were synthesised by Khaliq S. et al. (2022) [130] via Fischer esterification. The activity of the obtained molecules against Gram-positive and Gram-negative bacterial strains was investigated. All tested compounds exhibited concentration-dependent antibacterial activity against Gram-positive and Gram-negative bacterial strains. The compound 6-[(1-(4-aminobenzoyloxy)ethyl)]-3-[(5-(dimethylcarbamoyl)pyrrolidin-3-yl)thio]-4-methyl-7-oxo-1-azabicyclo[3.2.0]hept-2-ene-2-carboxylic acid (“M2”) (Figure 12 c) showed the highest potency, with inhibition zones ranging from 19 to 35 mm. In addition, three other compounds (“M3”, “M4”, and “M8”) also displayed promising broad-spectrum antibacterial activity [130]. The enhanced broad-spectrum antibacterial activity observed for the pyrrolidine-containing derivatives of this study marks the pyrrolidine ring’s significant role in antibacterial SAR studies. Because the pyrrolidine-containing side chain is conserved across these Meropenem derivatives, the observed differences in antibacterial activity are more plausibly associated with ester modification and its effects on lipophilicity, permeability, or chemical stability. Accordingly, this series illustrates the compatibility of the pyrrolidine-containing carbapenem scaffold with further prodrug-oriented optimisation rather than demonstrating a specific SAR contribution of the pyrrolidine ring.

#### 3.5.3. Tomopenem

Tomopenem, known as CS-023, is a new 1β-methylcarbapenem with a 5-substituted pyrrolidine-3-yl-thio group bearing an amidine moiety at the C-2 position (Figure 12d). A 2-guanidinoacetylamino pyrrolidine group at position 2 may be responsible for its activity against MRSA. This structural characteristic allows the antibiotic to promote the acylation reaction quickly [67].

The antibiotic Tomopenem was developed to treat nosocomial infections, including those caused by *Pseudomonas aeruginosa* and MRSA. It has a longer half-life comparable to that of other carbapenems and improved activity against Gram-positive and Gram-negative pathogens. Tomopenem had been in phase II clinical trials for bacterial infections, but its development has been discontinued [131,132]. The enhanced activity of Tomopenem against challenging pathogens, including MRSA, accentuates the value of the pyrrolidine scaffold. Tomopenem illustrates a more advanced use of the pyrrolidine scaffold, in which the ring serves as a three-dimensional linker that appropriately positions a cationic amidine-containing side chain, thereby supporting penetration and target engagement in Gram-negative bacteria.

### 3.6. Hybrid Molecules

Hybridisation is a widely applied medicinal chemistry approach that integrates two or more bioactive pharmacophores into a single molecular framework to achieve enhanced therapeutic efficacy. Although hybridisation is scientifically highly attractive, the successful clinical translation remains limited [133]. The incorporation of a pyrrolidine scaffold into hybrid molecules offers a valuable strategy for optimising antibacterial activity and molecular recognition of bacterial targets.

#### 3.6.1. Diazo Derivatives of 3,4-Dibenzo-Barrelene-Fused Pyrrolidine-2,5-Dione

**Figure 13:**
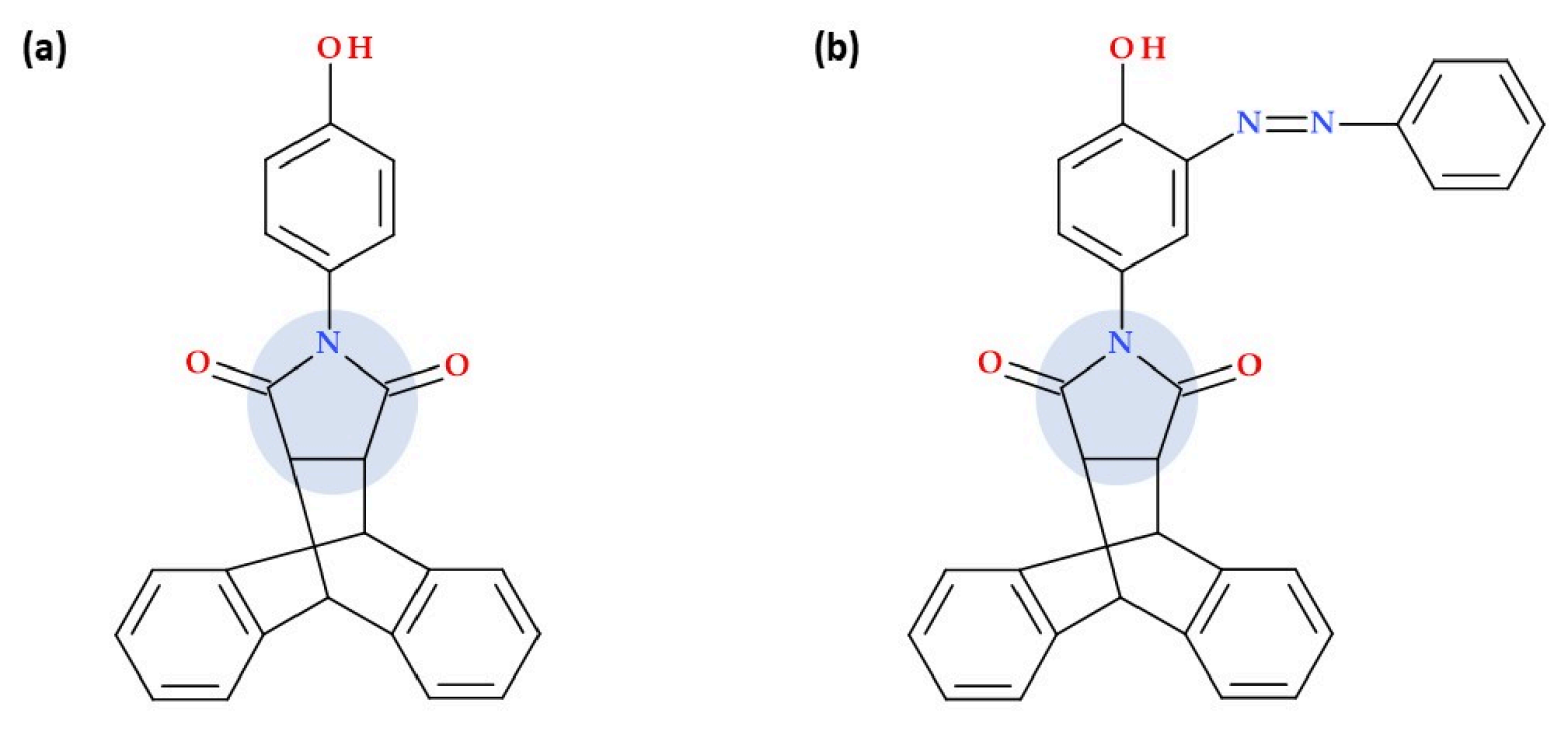
Molecular structure of two diazo derivatives of 3,4-dibenzo-barrelene-fused pyrrolidine-2,5-dione and their precursor: (**a**) 13-(4-hydroxyphenyl)-9,10-dihydro-9,10-ethanoanthracene-12,14-dicarboximide (“5”), (**b**) 13-(4-hydroxy-3-(phenyldiazenyl)phenyl)-9,10-dihydro-9,10-ethanoanthracene-12,14–dicarboximide (‘8”) [134].

Sopbué Fondjo E. et al. (2022) [134] reported new diazo derivatives of pyrrolidine-2,5-dione fused at the 3,4 positions with a dibenzobarrelene backbone obtained by coupling the precursor *N*-aryl succinimide with the aryldiazonium ion of aniline. The synthesised compounds are classified as hybrid molecules because they contain multiple pharmacologically relevant subunits (pyrrolidine-2,5-dione, dibenzobarrelene, phenol, and, for compound 8, an azo group) covalently linked within a single structure. The antimicrobial screens show that the three assessed compounds have moderate to low antibacterial and antifungal activity. Compounds 13-(4-hydroxyphenyl)-9,10-dihydro-9,10-ethanoanthracene-12,14-dicarboximide (“5”) and 13-(4-hydroxy-3-(phenyldiazenyl)phenyl)-9,10-dihydro-9,10-ethanoanthracene-12,14–dicarboximide (“8”) (Figure 13), exhibited moderate to weak antimicrobial activity against the tested bacterial and fungal strains, with MIC values ranging from 16 μg/mL to 256 μg/mL. Their efficacy was notably lower than that of the reference drugs Nystatin and Ciprofloxacin, which exhibited substantially stronger antimicrobial activity (MIC = 0.50–2 μg/mL and 0.50–16 μg/mL, respectively) [134]. The pyrrolidine-2,5-dione (succinimide) moiety was a key pharmacophoric scaffold in the synthesised hybrids, contributing to their antimicrobial activity and influencing their electronic properties via the nitrogen atom. Introduction of the pyrrolidine-derived fragment enhanced the antimicrobial activity relative to the precursor compound, supporting its role in the biological profile of these molecules.

#### 3.6.2. Oxadiazole/Pyrrolidine Hybrids

**Figure 14:**
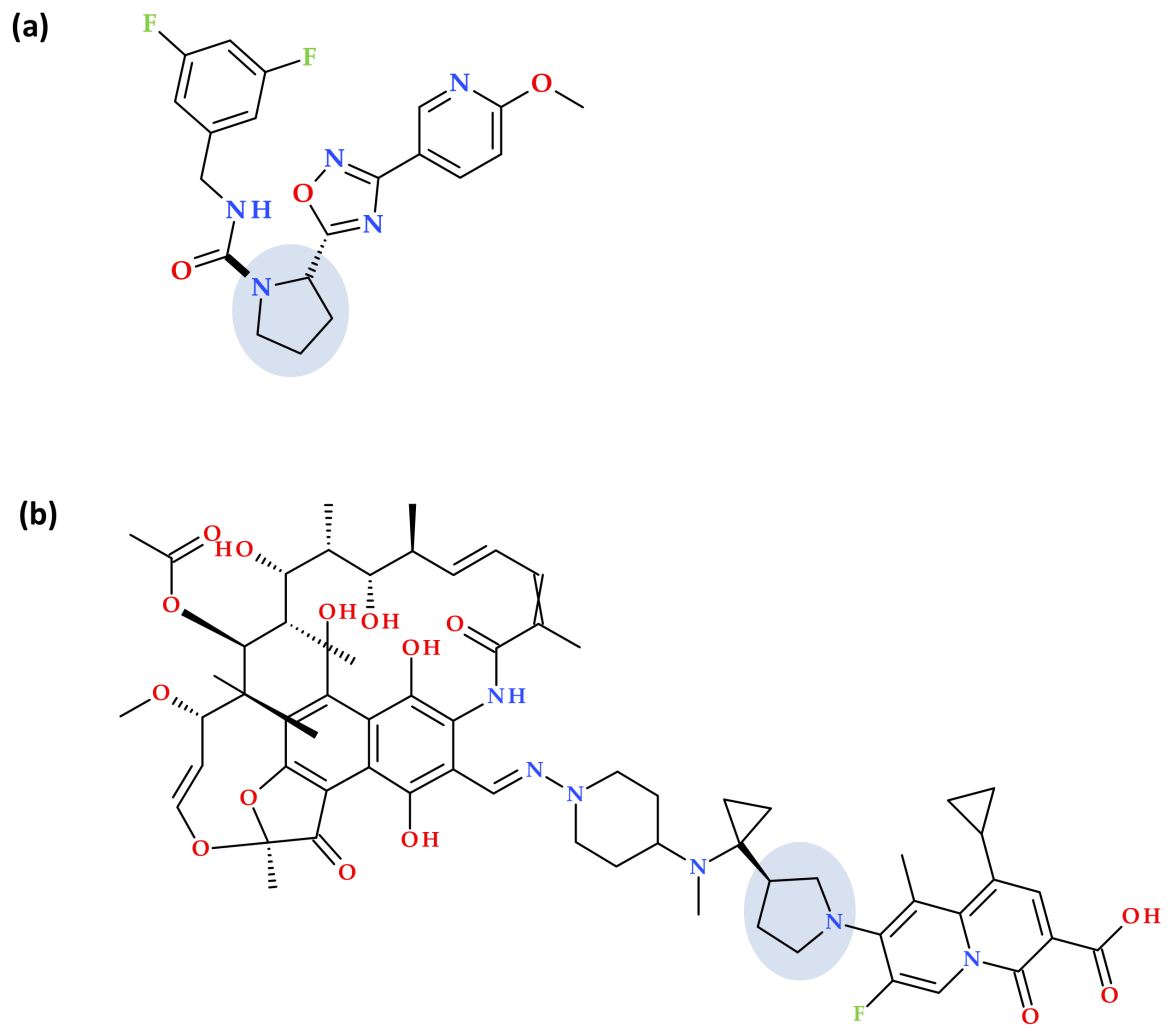
The molecular structure of (**a**) (*S*)-*N*-(3,5-difluorobenzyl)-2-[3-(6-methoxypyridin-3-yl)-1,2,4-oxadiazol-5-yl]pyrrolidine-1-carboxamide [135], and (**b**) Rifaquizinone (TNP-20292) [136,137,138], highlighting the pyrrolidine heterocycle.

A series of pyrrolidine compounds hybridised with a 1,2,4-oxadiazole moiety was synthesised by Frejat F.O.A. et al. (2022) [135] to develop molecules effective against DNA gyrase and topoisomerase IV enzymes. The DNA gyrase inhibitory assay results indicated two molecules with the most effective activity. Among the evaluated compounds, (*S*)-*N*-(3,5-difluorobenzyl)-2-[3-(6-methoxypyridin-3-yl)-1,2,4-oxadiazol-5-yl]pyrrolidine-1-carboxamide (“17”) (Figure 14a) showed the strongest inhibitory activity against *Escherichia coli* topoisomerase IV, comparable to that of Novobiocin. The compound also exhibited potent antibacterial activity against *Escherichia coli*, with an MIC of 55 ng/mL, surpassing Ciprofloxacin (MIC = 60 ng/mL). The combined docking and biological evaluation results identify this molecule as a promising lead candidate for the development of dual-target inhibitors of DNA gyrase and topoisomerase IV [135]. The results of this study demonstrate that incorporation of a pyrrolidine moiety can significantly contribute to the efficacy of inhibitors targeting bacterial DNA gyrase and topoisomerase IV.

#### 3.6.3. Rifaquizinone (TNP-2092)

Rifaquizinone (TNP-2092; formerly CBR-2092) is a rifamycin–quinolone hybrid antibacterial agent currently undergoing clinical evaluation. The molecule incorporates Rifamycin and quinolone pharmacophores within a single molecular framework, enabling dual inhibition of bacterial RNA polymerase and DNA gyrase/topoisomerase IV. Structurally, the quinolone-derived moiety is linked via a pyrrolidine-containing linker, highlighting the contribution of the pyrrolidine scaffold to the design of this promising hybrid antibacterial candidate (Figure 14b) [136,137,138]. Pyrrolidine derivatives were used as key starting materials in the synthesis of the quinolizinone core derived from the lead compound ABT-714 [136]. Subsequently, the optimised analogue, ABT-719, was synthesised at Abbott Laboratories and served as the quinolone-derived component in the development of hybrid antibacterial agents [139]. Building on this scaffold, Rifaquizinone was developed as a Rifamycin–quinolone hybrid antibiotic that combines two distinct antibacterial pharmacophores within a single molecular structure. Currently, Rifaquizinone is undergoing advanced clinical evaluation by TenNor Therapeutics (Suzhou, China) for the treatment of bacterial infections and is being investigated in both oral and intravenous formulations [127,140]. Although it has demonstrated promising efficacy and safety in clinical studies, the compound has not yet received regulatory approval.

### 3.7. Leopolic Acid A

**Figure 15:**
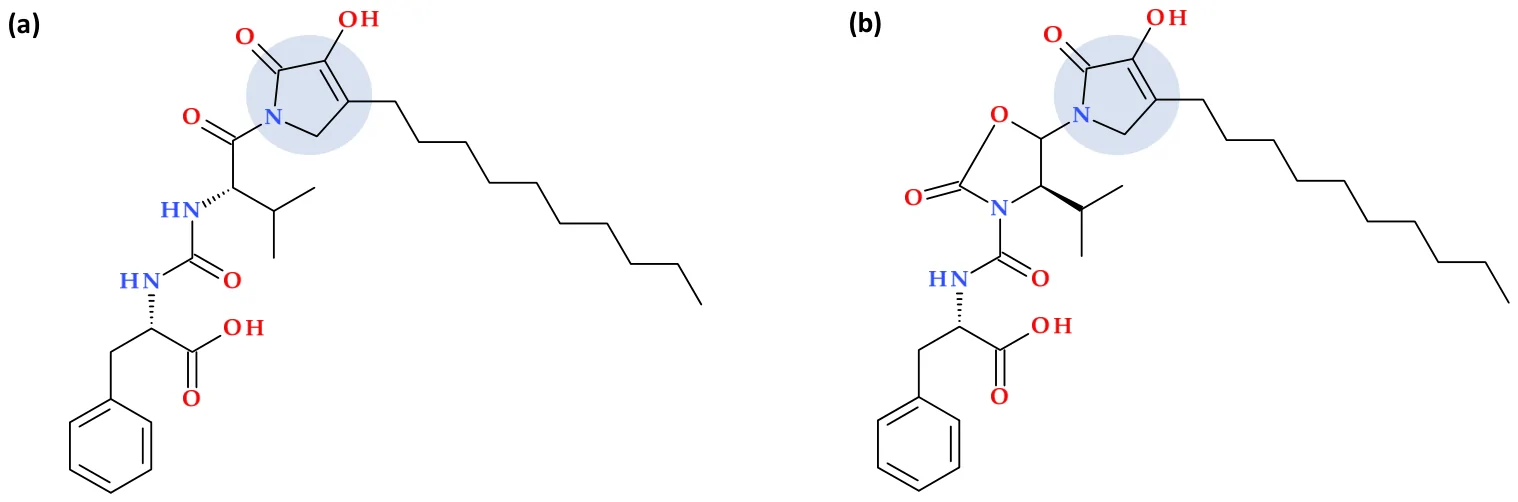
The molecular structure of (**a**) Leopolic acid A and (**b**) (2*S*,4′*S*,5′*R*) 2-{[5-(4-decyl-3-hydroxy-2-oxo-2,5-dihydro-1*H*-pyrrol-1-yl)-4-isopropyl-2-oxo-oxazolidine-3-carbonyl]amino}-3-phenylpropionic acid [142].

Leopolic acid A is a natural compound isolated from *Streptomyces* sp. found in the rhizosphere of *Juniperus excels*. Structurally, this compound contains a 2,3-pyrrolidine-dione headgroup (which predominantly exists as the enol tautomer) linked to an aliphatic chain appended to a urea-containing dipeptide consisting of *L*-valine and *L*-phenylalanine (Figure 15a). The molecular structure of Leopolic acid A can be a promising scaffold for the development of new antibacterial compounds. Leopolic acid A itself displayed modest antimicrobial activity against MRSA (MIC 32 μg/mL) [141].

Two studies reported synthesis and SAR studies of nine novel Leopolic acid A derivatives for their antimicrobial and antibiofilm properties. Leopolic acid A and the derivative (2*S*,4′*S*,5′*R*) 2-{[5-(4-decyl-3-hydroxy-2-oxo-2,5-dihydro-1*H*-pyrrol-1-yl)-4-isopropyl-2-oxo-oxazolidine-3-carbonyl]amino}-3-phenylpropionic acid (“17”) (Figure 15b) showed weak-to-moderate antibacterial activity against *Escherichia coli* (25 strains, MIC 128 μg/mL) and *Staphylococcus pseudintermedius* (20 strains, MIC 16 μg/mL), respectively. Compared with Leopolic acid A, the oxazolidinone analogue is more conformationally restricted and metabolically stable, owing to replacement of the flexible urea linker with a rigid cyclic carbamate, resulting in altered hydrogen-bonding properties. The obtained results suggest that these derivatives are valuable candidates for further development [141,142]. The antibacterial activity of Leopolic acid A and its derivatives highlights the pyrrolidine scaffold as a promising structural element for the design of new antibacterial and antibiofilm agents.

### 3.8. Spiropyrrolidine Derivatives

Nowadays, spiro compounds are attractive to synthetic chemists due to their rigid structures and facile incorporation into biological macromolecules.

**Figure 16:**
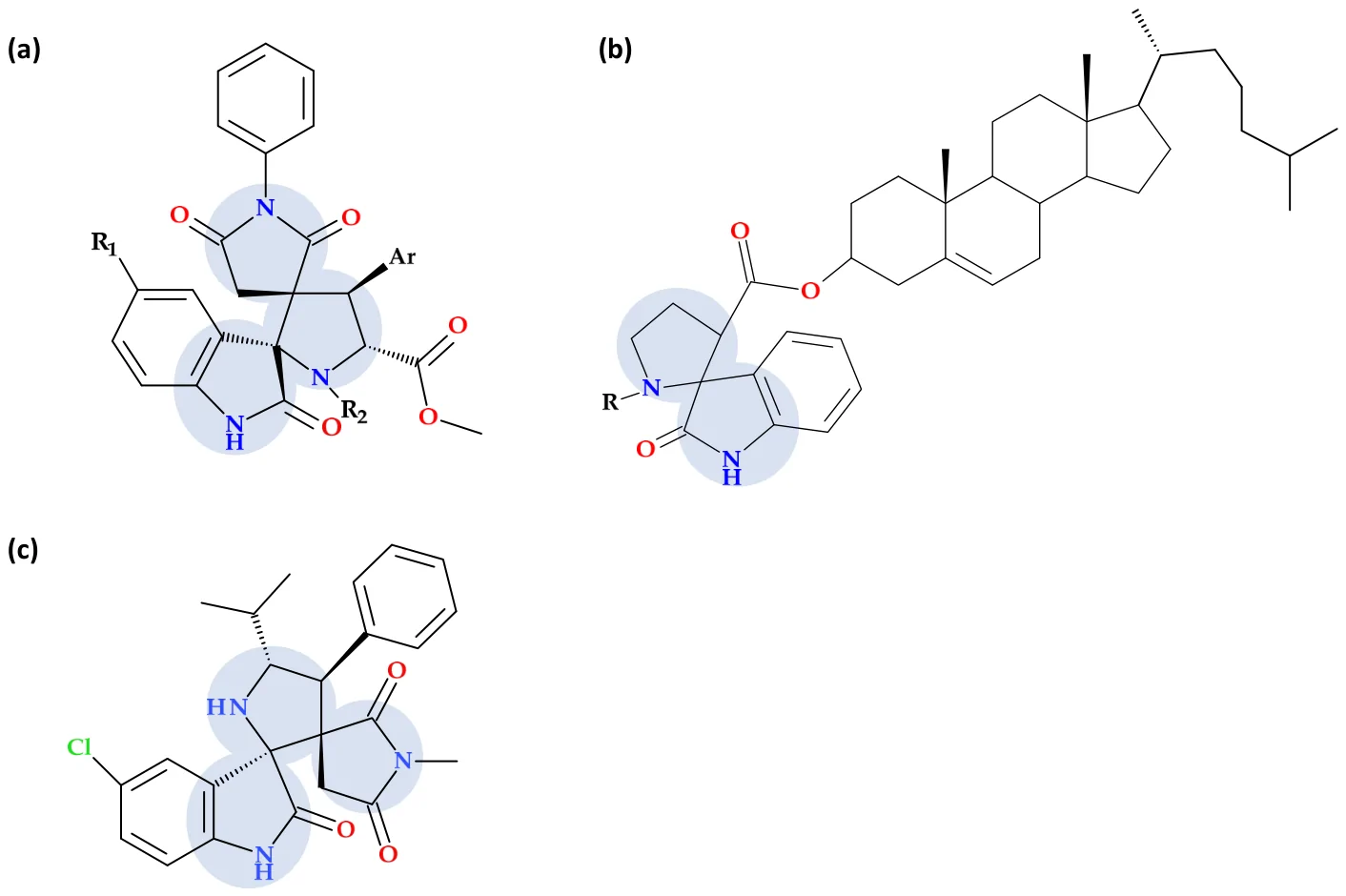
The molecular structure of some spiropyrrolidine derivatives: (**a**) spirooxindole pyrrolidine derivatives [143], (**b**) C3-*β*-cholesterol tethered spiropyrrolidine derivatives [144], (**c**) (2R*,3R*,4R*,5R*)-4-phenyl-spiro [2,3′]-5′-chloro-oxindolo-spiro [3,3″]-5-isopropylpyrrolidine-3-*N*-methylsuccinimide [145].

*Spirooxindole pyrrolidine derivatives*. A series of stereoselective spiro[pyrrolidine-2,3-oxindoles] (Figure 16a) was synthesised by Haddad S. et al. (2014) [143]. The compounds were tested in vitro for their antibacterial, antifungal, antimalarial and antitubercular activities. Antibacterial screening results indicated excellent activity compared to standard reference antibiotics. The nature and position of substituents on the pyrrolidine, isatin, and aryl moieties strongly influenced biological activity. Electron-withdrawing groups on the isatin scaffold enhanced antibacterial potency, particularly against *Escherichia coli*, while bromine substitution favoured activity against *Staphylococcus aureus* and *Streptococcus pyogenes*. Conversely, electron-donating methyl and methoxy substituents on the aromatic ring were associated with enhanced antitubercular activity. Three of the obtained compounds showed activity in vitro against *Mycobacterium tuberculosis* strain H37Rv with MIC values of 25–62 µg/mL [143]. The observed SAR further supports the pyrrolidine scaffold as a versatile pharmacophore for the design of new antibacterial compounds.

*C3-β-cholesterol tethered spiropyrrolidines*. New C3-β-cholesterol tethered spiropyrrolidines/pyrrolizidines (Figure 16b) were synthesised by Periyasami G. et al. (2019) [144]. A stereocycloaddition and dipolar cycloaddition reaction between the dipolarophile C3-*β*-cholesterolacrylate and azomethidine ylides yielded the desired compounds as single isomers. Concerning the antibacterial activity in vitro against four human pathogens, including *Vibrio cholerae*, *Proteus mirabilis*, *Micrococcus luteus*, and *Bacillus subtilis*, three compounds showed good antimicrobial activity at an MIC of 50 µg/mL. In silico docking studies demonstrated the role of the spiropyrrolidine moiety in inhibiting bacterial glucosamine-6-phosphate synthase (Glc-N-6-P) and, thus, in the antibacterial activity of the compounds [144]. The combined biological and computational results demonstrate the significant contribution of the spiropyrrolidine moiety to the antibacterial potential of these compounds.

*Other spiropyrrolidine derivatives*. A series of 17 spiropyrrolidine derivatives was obtained by Askri S. et al. (2022) [145] through three-component cascade reactions of (*E*)-3-arylidene-1-methyl-pyrrolidine-2,5-diones, *L*-valine and various isatin derivatives. The antibacterial and antifungal properties of the synthesised *N*-heterocyclic compounds were investigated in vitro. Several derivatives exhibit good activity comparable to that of the standard drugs (Amphotericin B and Tetracycline) when tested against the four bacterial strains (*Staphylococcus aureus*, *Micrococcus luteus*, *Escherichia coli*, and *Pseudomonas aeruginosa*). Among the seventeen pentacyclic spiro[oxindole-2,3′-pyrrolidine] derivatives tethered with succinimide scaffolds, the compound (2R*,3R*,4R*,5R*)-4-phenyl-spiro [2,3′]-5′-chloro-oxindolo-spiro [3,3″]-5-isopropylpyrrolidine-3-*N*-methylsuccinimide (“5a”) (Figure 16c) emerged as a potential lead antibacterial candidate. The derivative exhibited the highest activity against *Staphylococcus aureus* ATCC 25923, with an MIC of 3.9 μg/mL, an MBC of 31.5 μg/mL, and an inhibition zone of 31 mm. Molecular docking studies further supported its antibacterial potential by revealing favourable interactions with glucosamine-6-phosphate synthase and methionyl-tRNA synthetase [145]. The combined in vitro and molecular docking results demonstrate the key role of the pyrrolidine-containing scaffold in the development of potent antibacterial compounds.

### 3.9. Structurally Diverse Pyrrolidine-Based Antibacterial Chemotypes

Numerous other pyrrolidine derivatives with diverse structural features have been reported as antibacterial candidates. Modification of the pyrrolidine nucleus by introducing various pharmacophoric groups has yielded compounds with broad-spectrum antimicrobial activity. The most relevant derivatives will be discussed further.

#### 3.9.1. Hydroxy Pyrrolidine Derivatives

**Figure 17:**
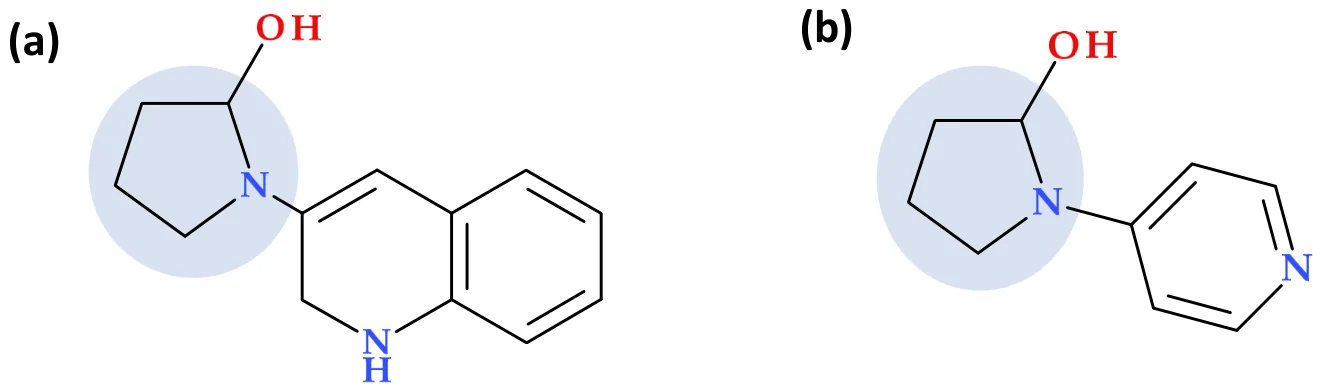
Molecular structure of the 2-hydroxy pyrrolidine derivatives: (**a**) 1-(quinolin-3-yl)pyrrolidin-2-ol, and (**b**) 1-(pyridin-4-yl)pyrrolidin-2-ol [146].

A new series of various 2-hydroxy pyrrolidine derivatives was synthesised by Suresh M. et al. (2016) [146]. The most potent compound was 1-(quinolin-3-yl)pyrrolidin-2-ol (“P7”) (Figure 17a), which exhibited inhibition zones of 28 ± 0.14 mm against *Escherichia coli* (MTCC 78) and 23 ± 0.14 mm against *Klebsiella pneumoniae*, corresponding to 100% relative inhibitory zones. The compound 1-(pyridin-4-yl)pyrrolidin-2-ol (“P3”) (Figure 17b) showed moderate antibacterial activity, with relative inhibitory zones of 56% against *Escherichia coli* (MTCC 78) and 80% against *Klebsiella pneumoniae* [146]. The pyrrolidine ring was used as a heterocyclic scaffold, enabling the incorporation of diverse heteroaromatic pharmacophores. The compound 1-(quinolin-3-yl)pyrrolidin-2-ol exhibited broad-spectrum antibacterial activity, highlighting the contribution of the pyrrolidine to the biological activity.

#### 3.9.2. Pyrrolidine Acetamide Derivatives

**Figure 18:**
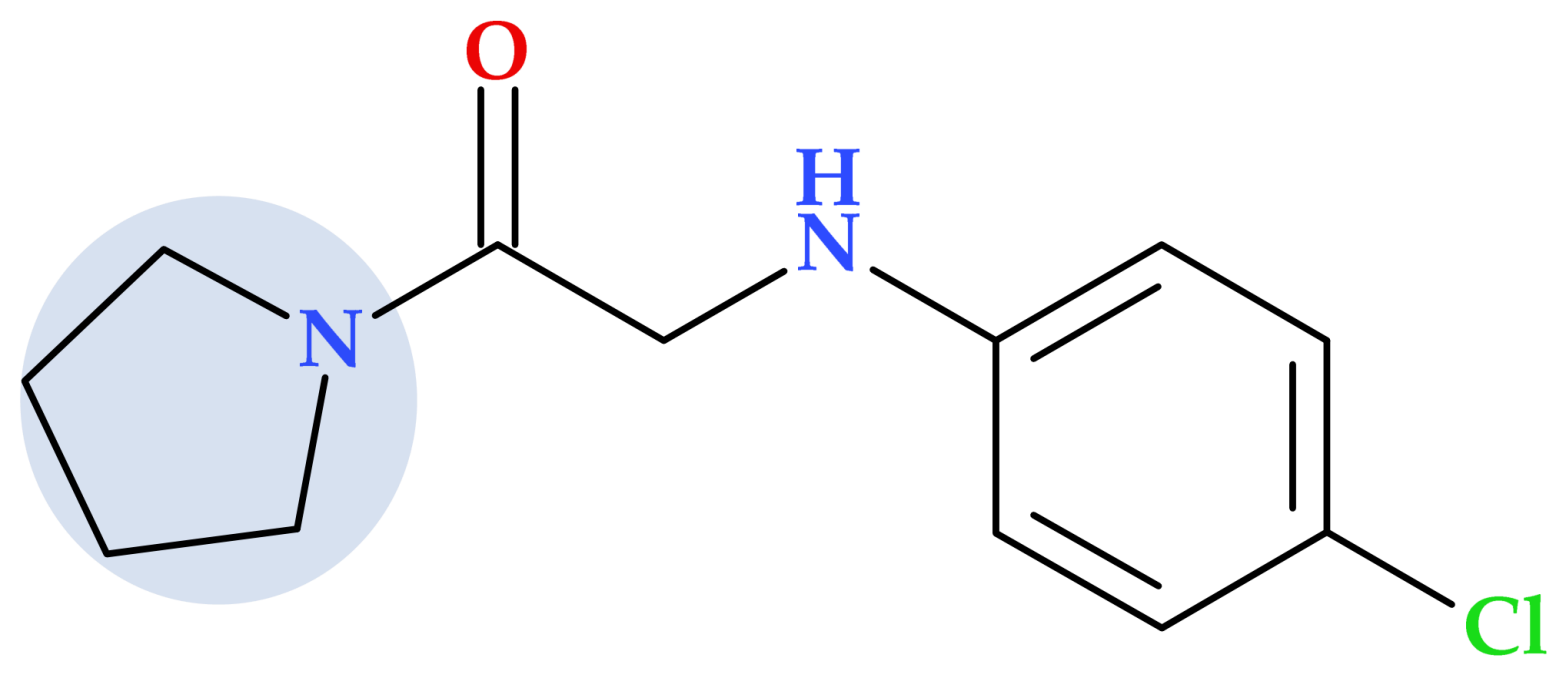
Molecular structure of the compound ((4-chlorophenyl)amino)-1-(pyrrolidin-1-yl)ethan-1-one [147].

In the study of Alsamarrai A.S.H. and Abdulghani S.S. (2021) [147], seven novel acetamide derivatives of primary and secondary amines and para-toluene sulphinate sodium salt were synthesised. The antibacterial activity of the pyrrolidine acetamide derivatives has been evaluated in vitro against one Gram-positive and two Gram-negative bacterial species (*Streptococcus pyogenes*, *Escherichia coli* and *Proteus mirabilis*). Among them, two compounds exhibited the most potent antibacterial activity. The compound ((4-chlorophenyl)amino)-1-(pyrrolidin-1-yl)ethan-1-one (“22”) (Figure 18) inhibited *Escherichia coli*, *Proteus mirabilis*, and *Streptococcus pyogenes* at MIC values of 12.5 μg/mL. The second compound (“24”), a piperidine analogue of (“22”), showed activity at 37.5 μg/mL against *Escherichia coli* and *Proteus mirabilis*, and at 12.5 μg/mL against *Streptococcus pyogenes* [147]. The superior antibacterial activity of the pyrrolidine acetamide derivative versus the piperidine analogue suggests that the pyrrolidine ring is a valuable scaffold. Also, the presence of the para-chlorophenyl-substituted acetamide moiety appears to be an essential structural feature contributing to antimicrobial potency.

#### 3.9.3. Pyrrolidine-Based Calixarene Derivatives

**Figure 19:**
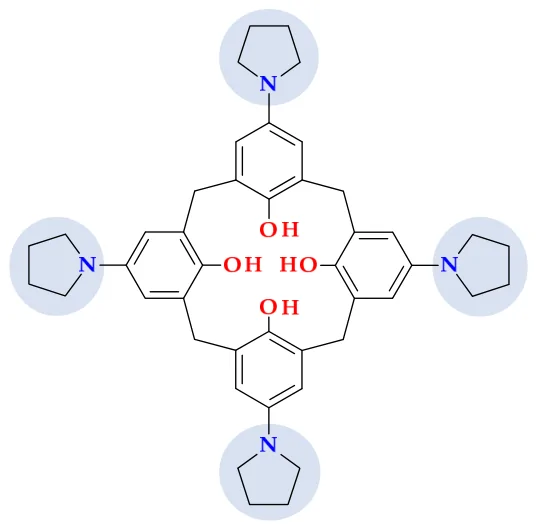
Molecular structure of pyrrolidine-functionalized calix[4]arene (CAP3), highlighting the pyrrolidine moieties associated with the antibacterial activity [148].

Muneer et al. (2017) [148] conducted a study on the synthesis, antibacterial and antifungal properties of calix[4]arene appended with pyrrolidine at the upper rim (CAP3). The new compounds showed antimicrobial activity against *Escherichia coli*, *Staphylococcus aureus*, and *Streptococcus viridans*. CAP3, depicted as a 5,11,17,23-tetrakis(*N*-pyrrolidinomethyl)-25,26,27,28-tetrahydroxycalix[4]arene (Figure 19), exhibited good antibacterial activity against *Streptococcus viridans* with MIC values of 0.58 and 1.17 mg/mL. The SAR study was evidenced by the presence of pyrrolidine substituents on the phenyl rings of the calix[4]arene [148].

#### 3.9.4. Pyrrolidine Carboxamide Derivatives

**Figure 20:**
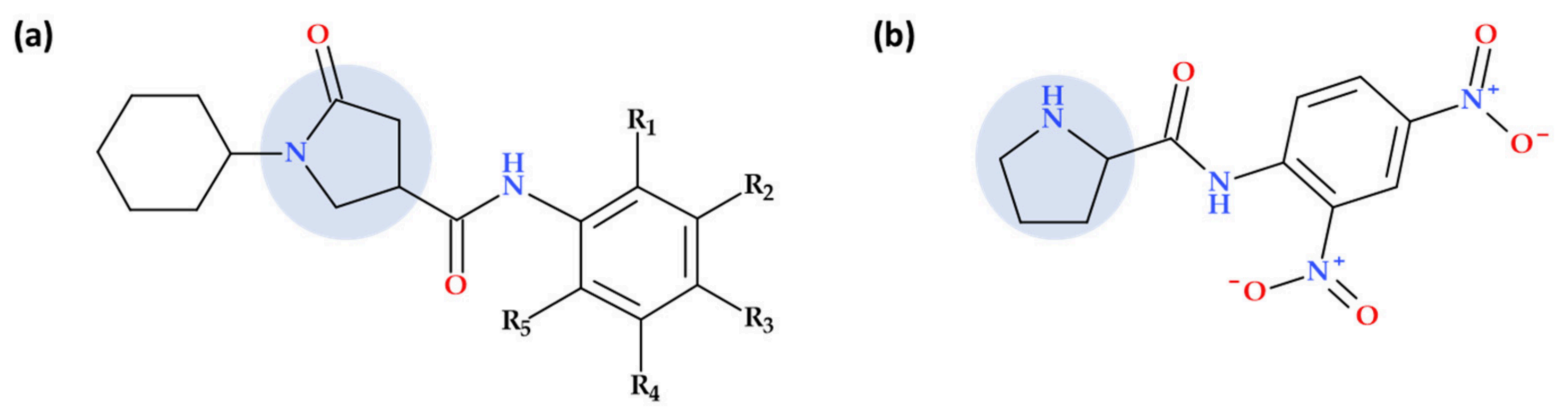
Molecular structure of (**a**) pyrrolidine carboxamide derivatives [149], and (**b**) *N*-(2′,4′-dinitrophenyl)pyrrolidine-2-carboxamide [150].

Pyrrolidine carboxamide inhibitors are a group of compounds that exhibit inhibitory activity against enoyl-acyl carrier protein reductase of *Mycobacterium tuberculosis*. A QSAR study by Kouassi A.F. et al. (2015) [149] highlighted that the best InhA inhibitors are the pyrrolidine carboxamide family (PCAMs). Following this study, several pyrrolidine carboxamide derivatives (Figure 20a) were synthesised and evaluated for their ADME profiles and potency. Several compounds showed favourable inhibitory activity against the target enzyme at low nanomolar concentrations, ranging from 10.5 nM to 4.5 nM, and may represent new leads for the development of antituberculosis drugs [149]. The pyrrolidine ring played an essential role in this class of InhA inhibitors, serving as a rigid heterocyclic scaffold that positions the carboxamide moiety and other pharmacophoric groups for interaction with the enzyme’s active site. The favourable potency and ADME profiles of the synthesised derivatives highlight the importance of the pyrrolidine in the design of new antitubercular candidates.

Antimicrobial peptides that bind to the bacterial protein DnaK are attracting increasing interest because they exhibit inhibitory effects on bacteria and other pathogens. Odusami J.A. et al. (2020) [150] reported the synthesis of novel N-(2′-nitrophenyl)pyrrolidine-2-carboxamides intended to resemble antimicrobial peptides. The introduction of diverse N′-substituents provided a suitable framework for evaluating the impact of conformational flexibility on the antibacterial properties of the synthesised compounds. With MIC values ranging from 15.6 to 512 μg/mL, promising antibacterial activity results were observed against five Gram-positive and five Gram-negative pathogens. The compound *N*-(2′,4′-dinitrophenyl)pyrrolidine-2-carboxamide (Figure 20b) exhibited a low MIC of 15.6 μg/mL against Gram-positive *Staphylococcus aureus* [150]. Also, in this study, the pyrrolidine ring acts as a structural surrogate of proline, the characteristic amino acid of proline-rich antimicrobial peptides. Through this proline-mimetic function, the pyrrolidine scaffold provides conformational restriction and contributes to the antibacterial activity of the synthesised *N-*(2-nitrophenyl)pyrrolidine-2-carboxamides.

#### 3.9.5. Pyrrolidine-2-One Derivatives

**Figure 21:**
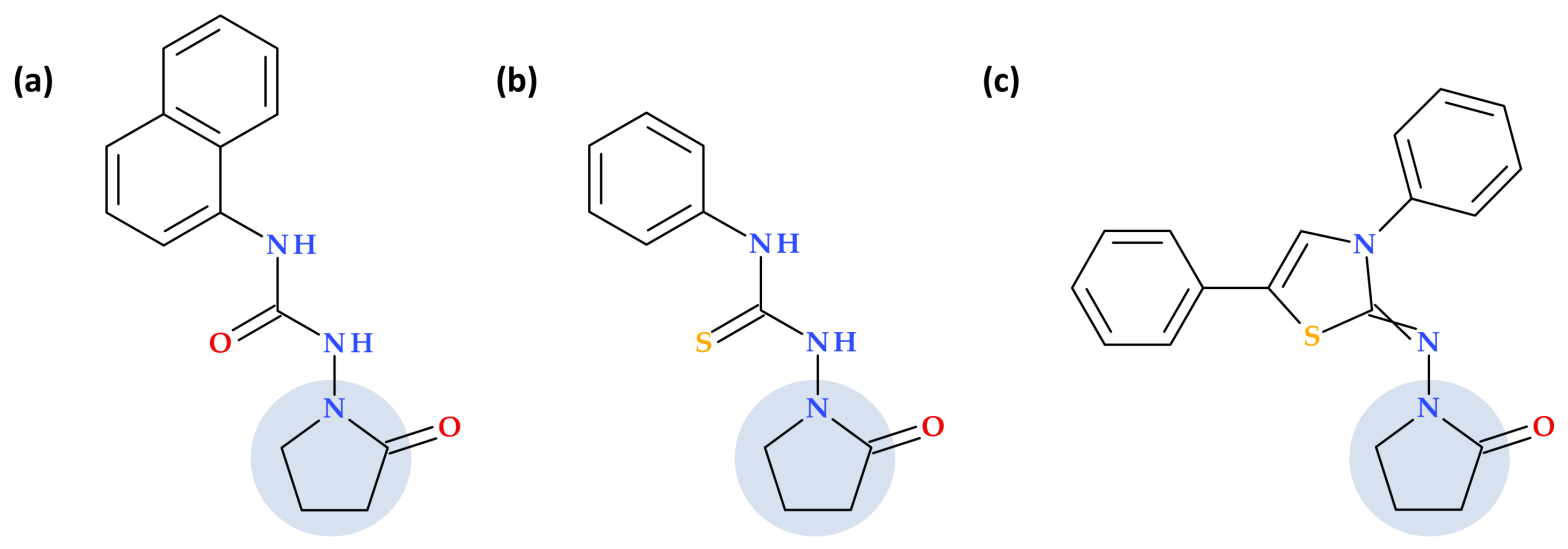
Molecular structure of (**a**) 1-naphthalen-1-yl-3-(2-oxopyrrolidin-1-yl)urea, (**b**) 1-(2-oxopyrrolidin-1-yl)-3-phenylthiourea, and (**c**) 1-{[(2*Z*)-3,5-diphenyl-1,3-thiazol-2(3*H*)-ylidene]amino}pyrrolidin-2-one [151].

Betti N.A. et al. (2020) [151] synthesised 15 novel pyrrolidine-2-one derivatives and evaluated their antibacterial activity against *Escherichia coli* and *Staphylococcus aureus* using the agar well diffusion method. The compounds were prepared through lactamization of γ-butyrolactone followed by derivatisation reactions to generate Schiff bases, thioureas, thiazoles, and other pyrrolidine-containing structures. Among the synthesized pyrrolidine-2-one derivatives, 1-naphthalen-1-yl-3-(2-oxopyrrolidin-1-yl)urea (“N10”), 1-(2-oxopyrrolidin-1-yl)-3-phenylthiourea (“N11”), and 1-{[(2*Z*)-3,5-diphenyl-1,3-thiazol-2(3*H*)-ylidene]amino}pyrrolidin-2-one (“N12”) (Figure 21) exhibited the strongest antibacterial activity, producing inhibition zones of up to 22, 20, and 21 mm, respectively, showing activity comparable to the reference antibiotic Amoxicillin [151]. The study’s results indicate that the pyrrolidine-2-one scaffold is a promising scaffold for the development of new antibacterial agents.

#### 3.9.6. Pyrrolidine-2,3-Dione Derivatives

**Figure 22:**
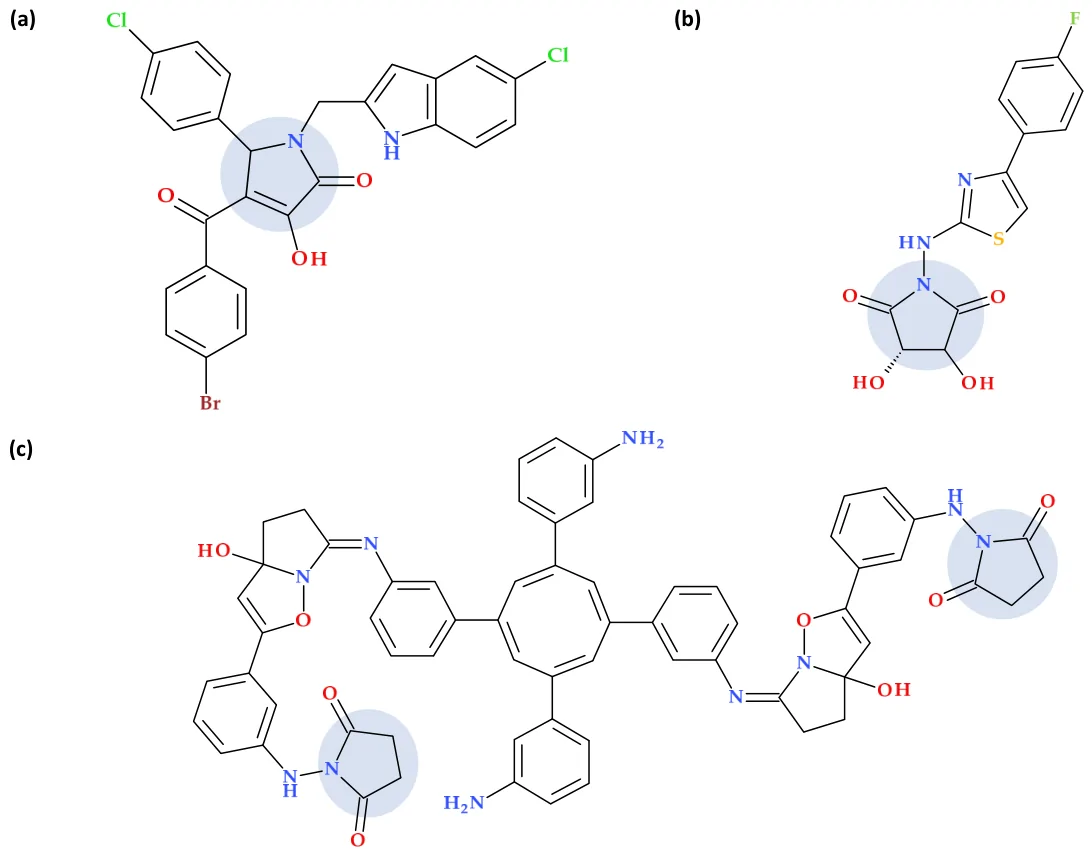
Molecular structure of pyrrolidine-2,3-dione and pyrrolidine-2,5-dione derivatives: (**a**) 3-(4-bromobenzoyl)-1-[(5-chloro-1*H*-indol-2-yl)methyl]-2-(4-chlorophenyl)-4-hydroxy-2*H*-pyrrol-5-one [152], (**b**) (3*S*)-1-[[4-(4-fluorophenyl)thiazol-2-yl]amino]-3,4-dihydroxy-pyrrolidine-2,5-dione [153], (**c**) 1-[3-[(6*Z*)-6-[3-[(1*E*,3*E*,5*E*,7*E*)-3,7-bis(3-aminophenyl)-5-[3-[(Z)-[2-[3-[(2,5-dioxopyrrolidin-1-yl)amino]phenyl]-3*a*-hydroxy-4,5-dihydropyrrolo[1,2-b]isoxazol-6-ylidene]amino]phenyl]cycloocta-1,3,5,7-tetraen-1-yl]phenyl]imino-3*a*-hydroxy-4,5-dihydropyrrolo[1,2-b]isoxazol-2-yl]anilino]pyrrolidine-2,5-dione [154].

More opportunities to inhibit PBP3 have appeared since the discovery of the pyrrolidine-2,3-dione class, which targets MDR bacterial strains. A series of pyrrolidine-2,3-diones was synthesised as novel non-beta-lactam inhibitors against PBP3 of the bacterium *Pseudomonas aeruginosa* by López-Pérez A. et al. (2021) [152]. The research identified two critical structural characteristics necessary for target inhibition: a 3-hydroxyl group (R_2_) and a heteroaryl group (R_1_) attached to the *N*-pyrrolidine-2,3-dione via a methylene linker. The results indicated that the compounds inhibited the growth of *Pseudomonas aeruginosa* and the efflux mutant, demonstrating a direct correlation between target inhibition and antibacterial activity. The most potent compound reported in the paper was a 5-chloro-indole pyrrolidine-2,3-dione derivative (“35”), namely 3-(4-bromobenzoyl)-1-[(5-chloro-1*H*-indol-2-yl)methyl]-2-(4-chlorophenyl)-4-hydroxy-2*H*-pyrrol-5-one (Figure 22a), with PBP3 IC_50_ ≈ 3 µM and among the best antibacterial activities in the series. MIC against *Pseudomonas aeruginosa* PAO1 (+4 µg/mL polymyxin B nonapeptide (PMBN)) was 3.13 µM, and MIC against *Pseudomonas aeruginosa* K28926 efflux mutant (+4 µg/mL PMBN) was 12.5 µM; MIC assays were performed in the presence of PMBN to facilitate the penetration of pyrrolidine-2,3-dione derivatives into *Pseudomonas aeruginosa* cells [152]. The study identified pyrrolidine-2,3-dione as an attractive lead scaffold for the development of next-generation PBP-targeting antibacterials to address antimicrobial resistance.

#### 3.9.7. Pyrrolidine-2,5-Dione Derivatives

Various pyrrolidine-2,5-dione derivatives have been synthesised and evaluated for their antibacterial activity against both Gram-positive and Gram-negative pathogens.

Kocabaş E. et al. (2021) [153] reported the synthesis of thiazole-based pyrrolidine derivatives and their evaluation as potential antibacterial agents. Of the twelve synthesized compounds, the compound (3*S*)-1-[[4-(4-fluorophenyl)thiazol-2-yl]amino]-3,4-dihydroxy-pyrrolidine-2,5-dione (“11”) (Figure 22b) exhibited antibacterial activity in an agar well diffusion assay, producing inhibition zones of 21.70 ± 0.36 mm against *Bacillus cereus* and 30.53 ± 0.42 mm against *Staphylococcus aureus* at 400 μg/well; no activity was observed against *Escherichia coli* or *Salmonella typhimurium* [153]. The combination of the thiazole pharmacophore with the 3,4-dihydroxypyrrolidine-2,5-dione scaffold appears to enhance antibacterial potency. SAR analysis of the thiazole-based pyrrolidine-2,5-dione series revealed that only the 4-fluorophenyl-substituted compound (“11”) exhibits notable antibacterial activity, whereas the bromo-,chloro-, nitro-, and coumarin-containing analogues were inactive. The para-fluoro substituent clearly promotes antibacterial efficacy, while the pyrrolidine-2,5-dione scaffold serves as the common bioactive scaffold of the series.

Some pyrrolidine-2,5-dione derivatives from N-hydroxysuccinimide were prepared by Lauro F.-V. et al. (2022) [154]. The biological activity of the synthesised compound against some strains of Gram-negative and Gram-positive bacteria was evaluated. According to the results, the three compounds inhibit the growth of *Escherichia coli* and *Klebsiella pneumoniae*, suggesting they may be effective antibacterials against Gram-negative bacteria. The chemical names of the three compounds evidenced in the study are:

• 1-[3-[(6*Z*)-6-[3-[(1*E*,3*E*,5*E*,7*E*)-3,7-bis(3-aminophenyl)-5-[3-[(Z)-[2-[3-[(2,5-dioxopyrrolidin-1-yl)amino]phenyl]-3*a*-hydroxy-4,5-dihydropyrrolo[1,2-b]isoxazol-6-ylidene]amino]phenyl]cycloocta-1,3,5,7-tetraen-1-yl]phenyl]imino-3*a*-hydroxy-4,5-dihydropyrrolo[1,2-b]isoxazol-2-yl]anilino]pyrrolidine-2,5-dione (“6”);
• 1-[3-[(6*Z*)-6-[3-[3-(3-aminophenyl)cyclobuta-1,3-dien-1-yl]phenyl]imino-3*a*-hydroxy-4,5-dihydropyrrolo[1,2-b]isoxazol-2-yl]anilino]pyrrolidine-2,5-dione (“7”);
• 1-[3-[(6*Z*)-6-[3-[3-[(3,4-dimethoxyphenyl)methyl]cyclobuten-1-yl]phenyl]imino-3*a*-hydroxy-4,5-dihydropyrrolo[1,2-b]isoxazol-2-yl]anilino]pyrrolidine-2,5-dione (“8”).

These three compounds exhibited comparable antibacterial activity against the Gram-negative pathogens *Escherichia coli* and *Klebsiella pneumoniae*, with mean MIC values ranging from 0.46 to 0.50 mg/mL. Among them, compound (“6”) (Figure 22c) showed the most favourable antibacterial profile and was identified as the lead derivative of the series [154]. In the molecular structure of the compound (“6”), the pyrrolidine-2,5-dione ring contributes to target recognition via its carbonyl groups and supports potent antibacterial activity against Gram-negative bacteria. The incorporation of this scaffold within the highly functionalized aminophenyl-substituted cyclooctatetraene system appears to contribute to the enhanced antibacterial activity.

#### 3.9.8. Pyrrolidine-3-Carbonitrile Derivatives

**Figure 23:**
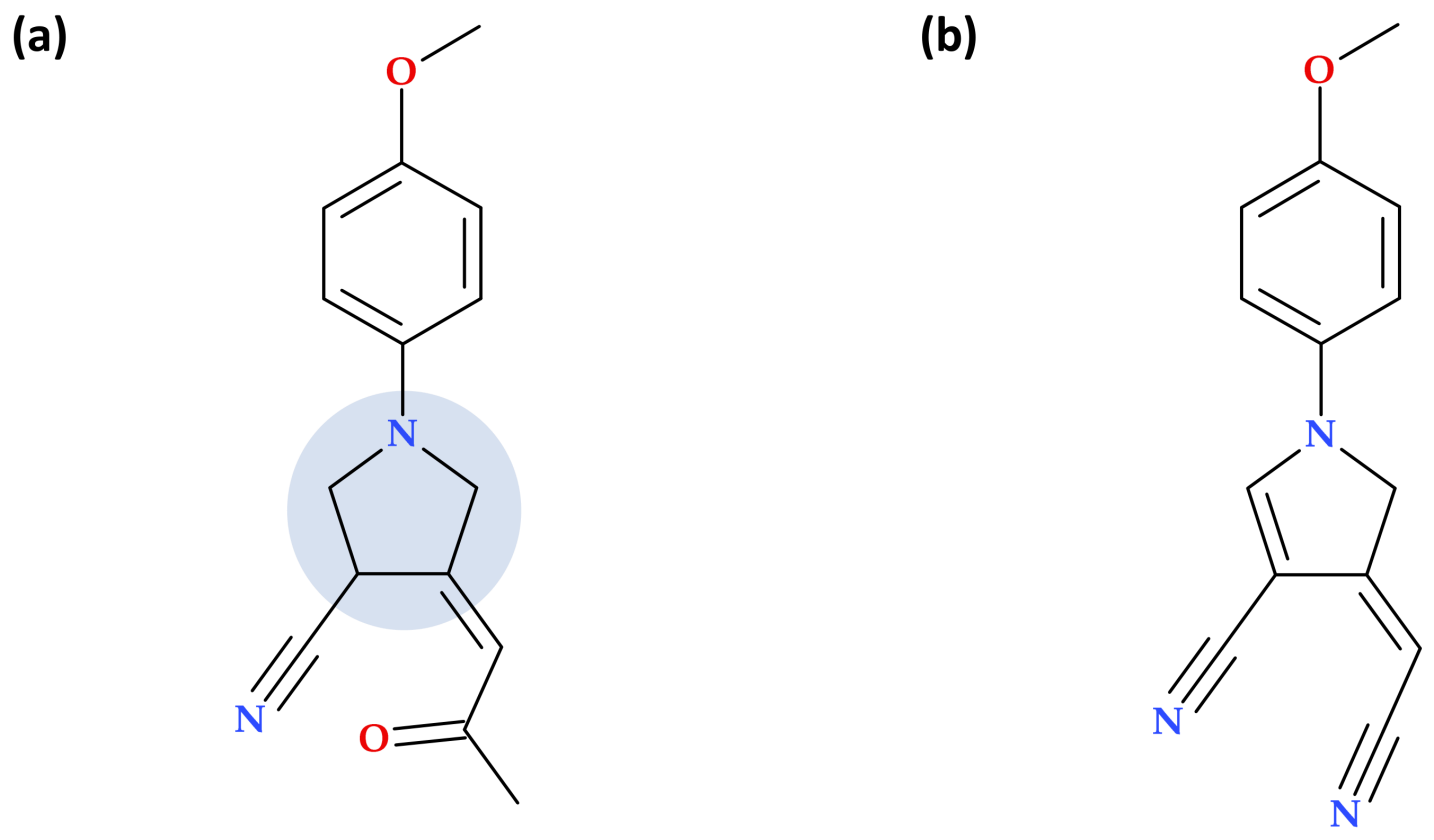
Molecular structure of compounds: (**a**) (*E*)-1-(4-methoxyphenyl)-4-(2-oxopropylidene)pyrrolidine-3-carbonitrile, and (**b**) (*E*)-4-(cyanomethylene)-1-(4-methoxyphenyl)-4,5-dihydro-1*H*-pyrrole-3-carbonitrile, the most active antimicrobial pyrrolidine-3-carbonitrile derivatives reported by El-Mansy et al. [155].

El-Mansy M. F. et al. (2018) [155] obtained new pyrrolidine-3-carbonitrile derivatives from the reactions of 1-(4-methoxyphenyl)-4-oxopyrrolidine-3-carbonitrile with stabilised phosphorus ylides. The novel compounds demonstrated antimicrobial activity against Gram-positive and Gram-negative bacteria. Among the synthesised pyrrolidine-3-carbonitrile derivatives, compound (*E*)-4-(cyanomethylene)-1-(4-methoxyphenyl)-4,5-dihydro-1*H*-pyrrole-3-carbonitrile (“7c”) was the most promising antimicrobial agent, exhibiting an inhibition zone of 20 mm against *Escherichia coli*, comparable to that of the reference antibiotic (Cephradine). Also, the compound (*E*)-1-(4-methoxyphenyl)-4-(2-oxopropylidene)pyrrolidine-3-carbonitrile (“6d”) showed an inhibition zone of 32 mm against *Staphylococcus aureus*, slightly exceeding the value reported for Cephradine (30 mm) (Figure 23). The results demonstrated antibacterial activity comparable to that of the reference antibiotic [155]. Thus, the antimicrobial activity of the two pyrrolidine-3-carbonitrile derivatives confirms that pyrrolidine is a key pharmacophore in the design of new anti-infective agents.

#### 3.9.9. Pyrrolidine-Containing Murrayanine-Based Chalcone Compound

**Figure 24:**
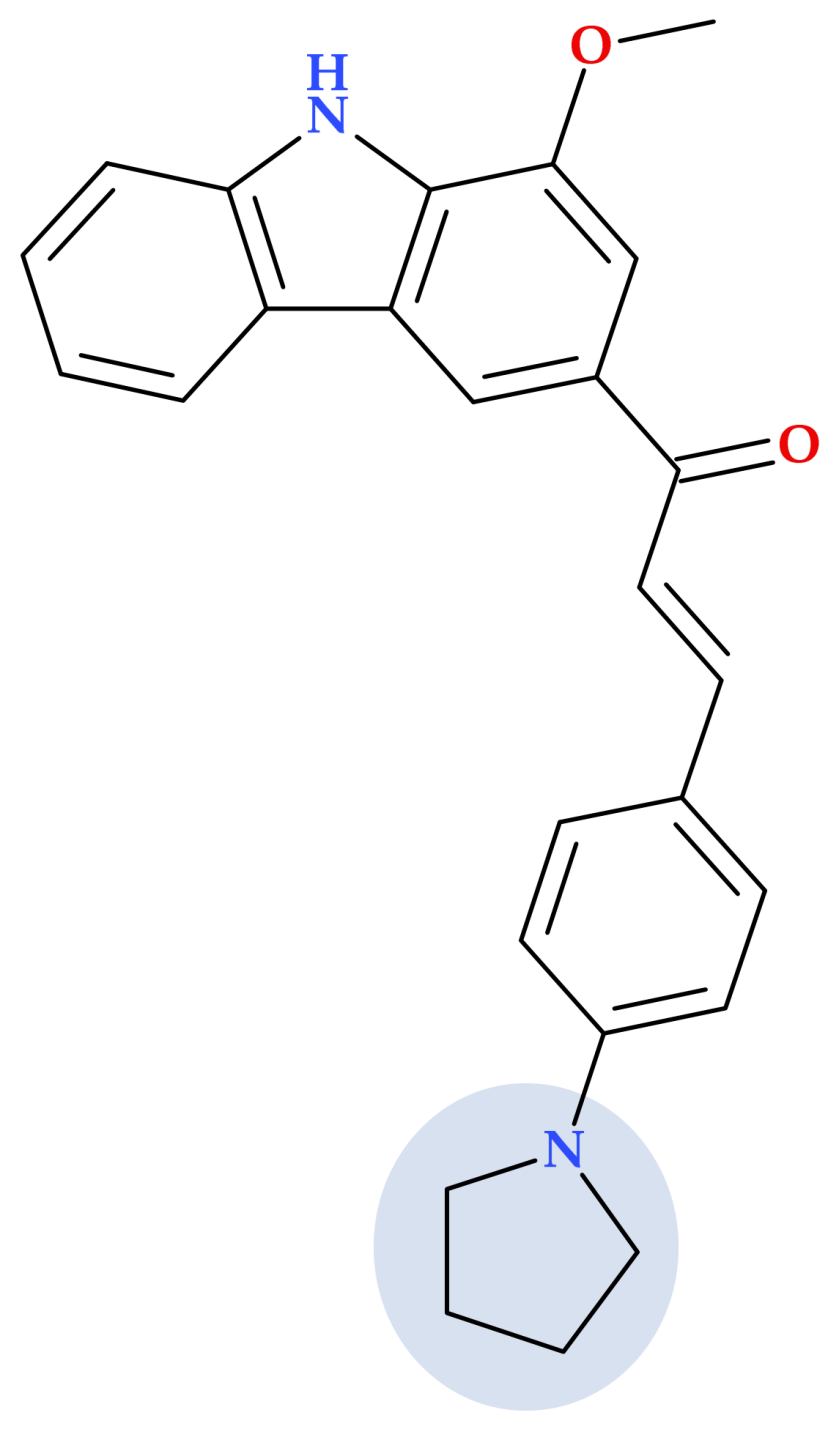
Molecular structure of the compound (*E*)-1-(1-methoxy-9*H*-carbazol-3-yl)-3-(4-(pyrrolidin-1-yl)phenyl)prop-2-ene-1-one [156].

The fusion of the natural compound murrayanin with pyrrolidine resulted in the chalcone compound (*E*)-1-(1-methoxy-9*H*-carbazol-3-yl)-3-(4-(pyrrolidin-1-yl)phenyl)prop-2-ene-1-one (Figure 24), which was tested against different pathogens. The novel pyrrolidine-containing chalcone exhibited noteworthy antibacterial activity, showing the highest activity against *Escherichia coli* (inhibition zone 23.97 ± 1.33 mm), followed by *Staphylococcus aureus* (18.76 ± 1.36 mm). Although its antibacterial potency was lower than that of the reference drug Ciprofloxacin, the benzylideneacetophenone scaffold incorporating murrayanine in ring A and pyrrolidine in ring B demonstrated promising antibacterial properties. It opened new perspectives for the development of novel antibacterial agents [156].

#### 3.9.10. Pyrrolidinone-Based Monocarbams

**Figure 25:**
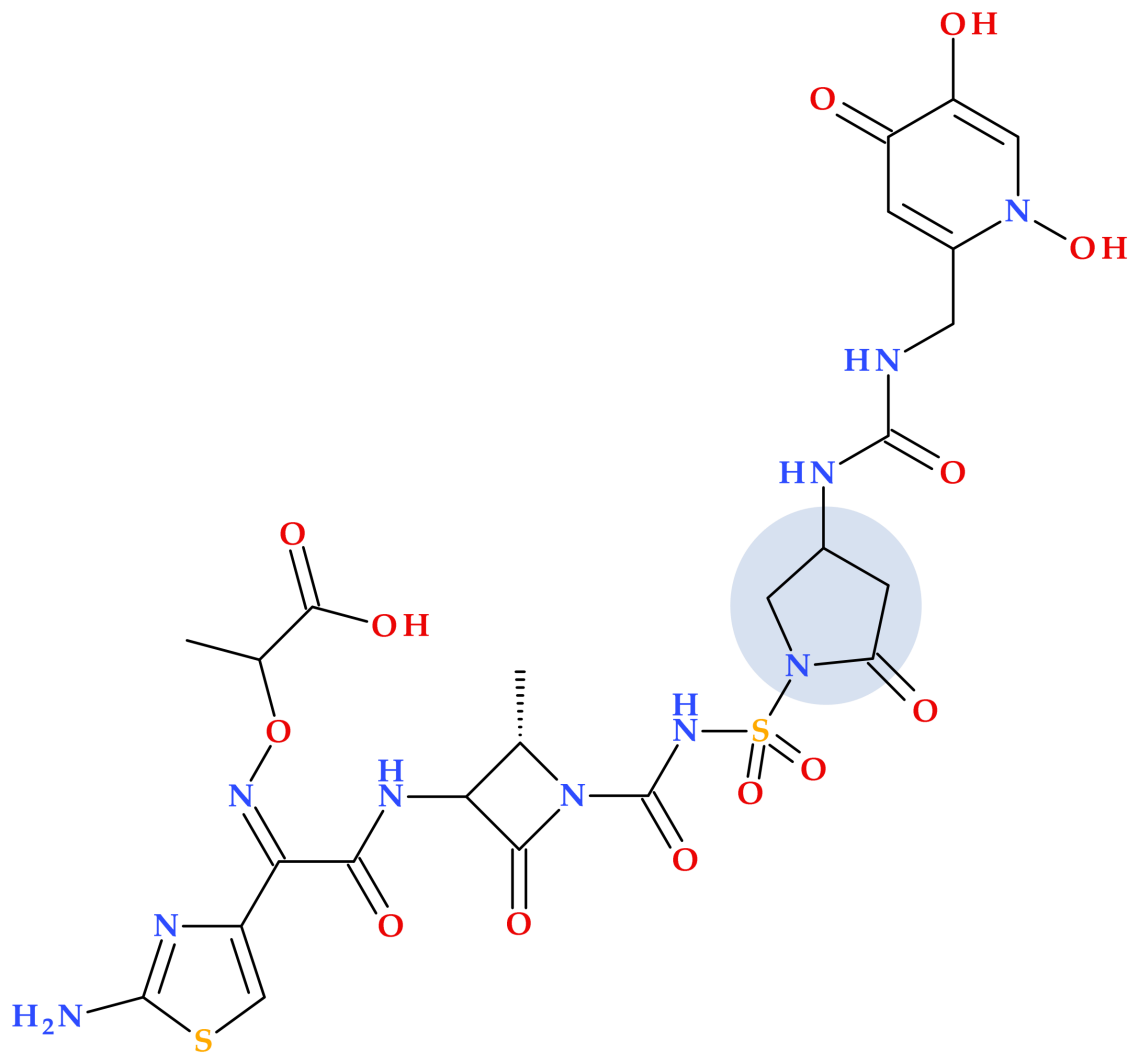
Molecular structure of compound 2-((((*Z*)-1-(2-aminothiazol-4-yl)-2-(((2*S*,3*S*)-1-((((*S*)-4-(3-((1,5-dihydroxy-4-oxo-1,4-dihydropyridin-2-yl)methyl)ureido)-2-oxopyrrolidin-1-yl)sulfonyl)carbamoyl)-2-methyl-4-oxoazetidin-3-yl)amino)-2-oxoethylidene)amino)oxy)-2-methylpropanoic acid [157].

A series of siderophore-conjugated pyrrolidinone-based monocarbams was developed as potential agents against multidrug-resistant *Pseudomonas aeruginosa*. These monocyclic beta-lactams exploit the bacterial iron-uptake system to enhance cellular penetration and retain stability against metallo-beta-lactamases. Systematic optimisation of the monocarbam linker, siderophore, and oxime moieties led to derivatives with potent antibacterial activity (*Pseudomonas aeruginosa* MIC90 = 2 μg/mL), favourable free-drug fractions (>20%), and excellent hydrolytic stability (t_1/2_ ≥ 100 h). Among them, the compound 2-((((*Z*)-1-(2-aminothiazol-4-yl)-2-(((2*S*,3*S*)-1-((((*S*)-4-(3-((1,5-dihydroxy-4-oxo-1,4-dihidropyridin-2-yl)methyl)ureido)-2-oxopyrrolidin-1-yl)sulfonyl)carbamoyl)-2-methyl-4-oxoazetidin-3-yl)amino)-2-oxoethylidene)amino)oxy)-2-methylpropanoic acid (“29”) (Figure 25) is as promising lead candidate due to its favourable pharmacokinetic properties and predicted in vivo efficacy [157]. The pyrrolidinone scaffold appears to have an essential role in the antibacterial profile of the highlighted compound, promoting efficient PBP3 inhibition and supporting the overall pharmacokinetic and stability characteristics of the molecule.

### 3.10. Pyrrolidine-Based Antibacterial Adjuvants and Resistance-Modifying Agents

#### 3.10.1. WCK-5153

**Figure 26:**
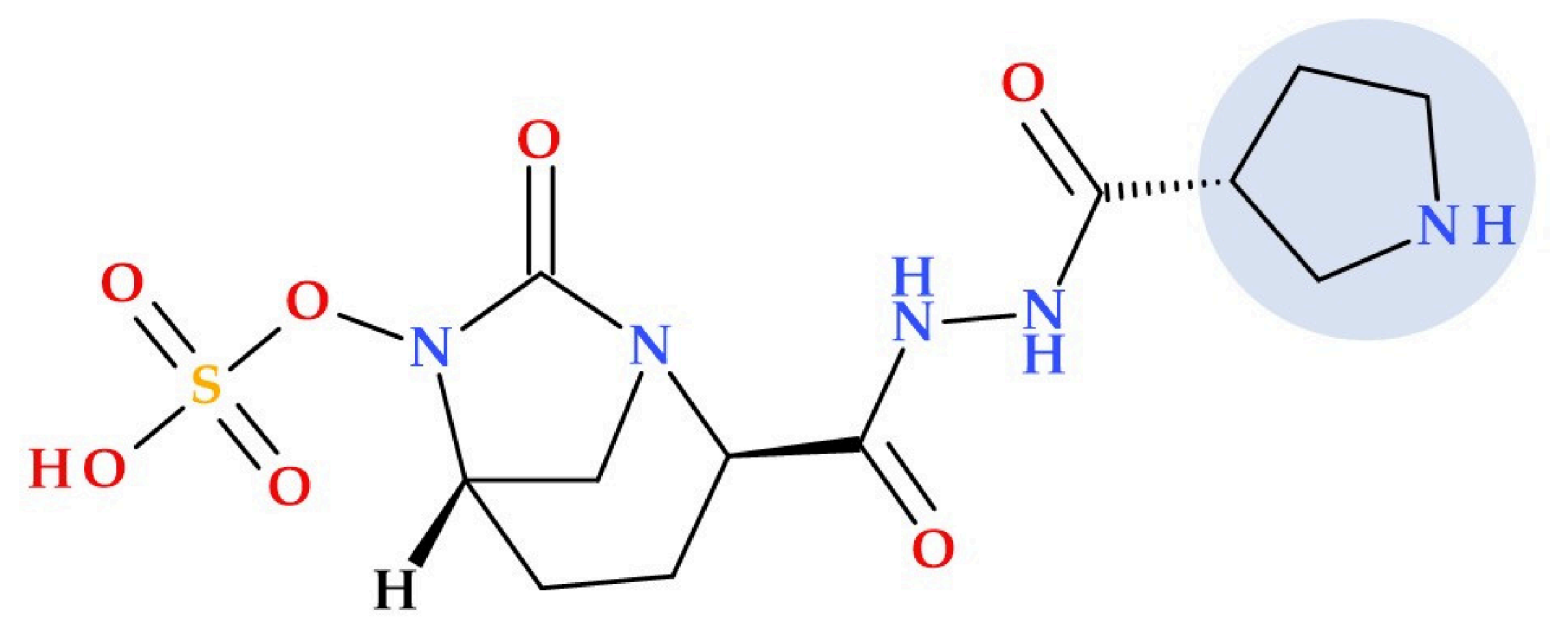
Molecular structure of WCK-5153 or [(2*S*,5*R*)-7-oxo-2-[[[(3*S*)-pyrrolidine-3-carbonyl]amino]carbamoyl]-1,6-diazabicyclo[3.2.1]octan-6-yl] hydrogen sulfate [158].

WCK-5153 is a novel pyrrolidine-containing beta-lactam enhancer belonging to the bicyclo-acyl hydrazide class (Figure 26) and one of the earliest representatives of a new generation of antibacterial adjuvants active against multidrug-resistant Gram-negative pathogens. The compound exhibits potent antibacterial activity against MDR *Pseudomonas aeruginosa* by selectively inhibiting PBP2. In vitro studies demonstrated MIC values ranging from 2 to 32 μg/mL, bactericidal activity in time-kill assays, and the ability to restore susceptibility to multiple beta-lactam antibiotics when combined. Notably, WCK-5153 enhanced the activity of antipseudomonal beta-lactams. It enabled complete eradication of certain metallo-beta-lactamase-producing high-risk clones, highlighting its potential as a promising adjunct for the treatment of multidrug-resistant Gram-negative infections [158]. The ability of WCK-5153 to restore beta-lactam susceptibility supports the pyrrolidine moiety as a key structural feature in the design of antibacterial adjuvants.

#### 3.10.2. Pyrrolidine Derivatives as Inhibitors of Bacterial Efflux Pumps

Gaucher B. and Dreier J. (2017) [159] reported a series of structurally diverse pyrrolidine derivatives as bacterial efflux pump inhibitors. The patented pyrrolidine derivatives comprise structurally diverse, highly substituted pyrrolidine-based molecules designed as bacterial efflux-pump inhibitors. The compounds were intended for use in combination with antimicrobial agents, especially antibiotics, to improve therapeutic efficacy against bacterial infections. Their mechanism of action involves inhibition of multidrug efflux systems, thereby enhancing antimicrobial susceptibility and mitigating both intrinsic and acquired resistance in pathogenic microorganisms [159]. The patent highlighted that the pyrrolidine scaffold is a promising platform for developing novel adjuvant agents to combat bacterial MDR.

Collectively, the compounds reviewed in this section demonstrate the broad adaptability of the pyrrolidine scaffold in antibacterial medicinal chemistry. The ring can act as a pharmacophoric element, a conformationally constrained linker, a carrier of cationic substituents, or a structural platform for combining multiple antibacterial motifs. The strength of the available evidence varies considerably. While several compounds exhibit encouraging MIC values or activity against resistant pathogens, many series remain supported only by inhibition-zone assays, limited strain panels, or preliminary patent data. Moreover, the specific contribution of the pyrrolidine moiety is not always distinguishable from that of the attached pharmacophores. Future studies should prioritise matched molecular-pair analyses, standardised MIC testing, cytotoxicity and selectivity assessment, mechanism-of-action studies, and in vivo validation to identify those pyrrolidine-containing chemotypes with genuine translational potential.

### 3.11. SAR Analysis of Pyrrolidine-Based Antibacterial Compounds

Analysis of the pyrrolidine-containing antibacterial compounds discussed in this review reveals several recurring SAR aspects. Although the investigated molecules belong to diverse chemical classes and frequently interact with different biological targets, the pyrrolidine scaffold consistently contributes to antibacterial activity by modulating molecular conformation, target recognition, membrane permeability, and physicochemical properties. Pyrrolidine is considered a useful heterocycle for modifying physicochemical properties and achieving optimal absorption, distribution, metabolism, and excretion (ADME)/Toxicological properties for drug candidates. The presence of a nitrogen atom can contribute to a molecule’s polarity by generating a dipole moment and a large polar surface area. The nitrogen atom also influences the lipophilicity of the heterocyclic nucleus [12,16,26].

#### 3.11.1. Positively Charged Pyrrolidinium Substituents in Gram-Negative Antibacterial Design

One of the clearest SAR features is the beneficial effect of quaternized pyrrolidinium substituents in Gram-negative antibacterial agents. The presence of a quaternary pyrrolidinium group can facilitate transport through porin channels of Gram-negative bacteria. The positively charged pyrrolidinium group may improve intracellular drug accumulation while maintaining stability against beta-lactamase hydrolysis, thereby enhancing antibacterial efficacy and supporting the development of novel antibacterial agents [68,69,160]. In cephalosporins such as Cefepime and Cefiderocol (Figure 3), the positively charged pyrrolidinium group contributes to the zwitterionic character of the molecule, facilitating penetration through outer-membrane porin channels and promoting intracellular accumulation. Simultaneously, these substituents improve stability against beta-lactamases while maintaining favourable affinity for PBPs, resulting in enhanced antibacterial activity against clinically important Gram-negative pathogens, including *Pseudomonas aeruginosa* and members of the Enterobacterales family [56,68,69,70,71,72,73,160].

Related design principles are observed in several carbapenems, in which ionisable pyrrolidine-containing side chains contribute to antibacterial spectrum, target engagement, and physicochemical properties. However, resistance to enzymatic degradation and renal dehydropeptidase hydrolysis also depends on additional structural features, including substitution at the C-1 position and the configuration of the carbapenem core.

#### 3.11.2. Pyrrolidine as a Conformationally Constrained Element in Target Recognition

The five-membered saturated pyrrolidine ring is a frequently used conformationally constrained scaffold that promotes productive interactions with biological targets. In ribosome-targeting antibiotics such as Clindamycin (Figure 5), Quinupristin (Figure 6), and Eravacycline (Figure 7), the pyrrolidine moiety contributes to the three-dimensional arrangement required for efficient target binding [93,94,95,97,108,112].

This feature is also evident among synthetic antibacterial candidates. Oxadiazole-pyrrolidine hybrids (Figure 14a) demonstrated potent inhibition of bacterial DNA gyrase and topoisomerase IV, while spiropyrrolidine derivatives (Figure 16) exhibited favourable interactions with glucosamine-6-phosphate synthase and methionyl-tRNA synthetase in molecular docking studies [135,144,145]. These observations suggest that rigid pyrrolidine-containing frameworks facilitate optimal positioning of key pharmacophoric groups within enzyme-binding pockets.

#### 3.11.3. Pyrrolidine-Containing Substituents Can Improve Activity Against Resistant Pathogens

Several successful antibacterial agents use pyrrolidine-containing modifications to overcome established resistance mechanisms. Eravacycline (Figure 7) provides a notable example. Introduction of the pyrrolidinoacetamide side chain at C-9 increases ribosomal binding affinity while simultaneously reducing susceptibility to efflux-mediated resistance, thereby preserving activity against tetracycline-resistant organisms, including MDR strains [108,112,113,114,115].

Similarly, Tomopenem (Figure 12d), WCK-5153 (Figure 26), and several beta-lactam enhancer candidates exploit pyrrolidine-containing substituents to improve interactions with PBPs and restore susceptibility to beta-lactam antibiotics in resistant Gram-negative pathogens [131,158]. These studies indicate that appropriate pyrrolidine substitution may contribute not only to antibacterial potency but also to resistance-modifying properties.

#### 3.11.4. Biological Activity Is Strongly Influenced by Substituents Attached to the Pyrrolidine Ring

Across numerous synthetic series, antibacterial activity was more strongly influenced by the nature of the attached substituents than by the pyrrolidine nucleus alone. For example, within the thiazole-based pyrrolidine-2,5-dione derivatives reported by Kocabaş E. et al. (2021) [153], only the 4-fluorophenyl-substituted analogue (Figure 22b) displayed notable antibacterial activity. In contrast, bromo-, chloro-, nitro-, and coumarin-containing analogues were considerably less active [153]. Also, the antibacterial activity of spiropyrrolidine derivatives (Figure 16) depended strongly on the electronic properties and substitution patterns of the aromatic groups present in the molecules [143,145].

Similar observations were reported for pyrrolidine acetamides (Figure 18), hydroxypyrrolidines (Figure 17), and pyrrolidine carboxamides (Figure 20a), in which antibacterial potency varied substantially depending on the nature and position of aromatic substituents [146,147,150]. Thus, the pyrrolidine ring should be considered primarily a valuable scaffold, with its antibacterial activity dependent on complementary pharmacophoric groups.

#### 3.11.5. Pyrrolidine Used as a Versatile Scaffold for Hybrid Antibacterial Design

Hybrid molecules containing pyrrolidine often exhibited enhanced antibacterial activity. Oxadiazole-pyrrolidine hybrids (Figure 14a), rifamycin-quinolone conjugates (Figure 14b), chalcone derivatives (Figure 24), and various spirocyclic systems demonstrated that the pyrrolidine nucleus can serve as a linker or structural platform to connect multiple pharmacophores [135,136,137,138,156]. Such architectures may enable the combination of complementary pharmacophoric elements and potentially support multitarget or resistance-modifying activity. However, superiority over the individual molecular fragments and the existence of genuinely complementary mechanisms should be confirmed experimentally.

#### 3.11.6. General SAR Conclusions

The available evidence indicates that the antibacterial activity of pyrrolidine-containing compounds arises from a combination of factors rather than the pyrrolidine ring alone. The most consistent SAR features indicate that:

• Quaternized pyrrolidinium groups enhance Gram-negative penetration and beta-lactamase stability [56,68,69,71,73,160];
• Conformationally constrained pyrrolidine scaffolds promote productive target interactions [12,16,93,94,97,108];
• Pyrrolidine-containing side chains can support the circumvention or modulation of selected resistance mechanisms, although these effects are strongly dependent on the complete molecular architecture [108,112,113,114,115,158];
• Antibacterial potency depends strongly on the identity, position, stereochemistry, and electronic properties of substituents attached to the pyrrolidine-containing framework [143,144,145,147,150,153];
• Pyrrolidine could be considered an effective scaffold for the design of hybrid antibacterial agents and resistance-modifying compounds [135,136,156,158,159].

All these aspects collectively support the classification of pyrrolidine as a valuable scaffold for antibacterial drug discovery and guide the rational design of next-generation pyrrolidine-based antibacterial agents [16,24]. However, its value does not arise from the heterocycle in isolation, but from its capacity to organise pharmacophoric groups in three-dimensional space, modulate ionisation and physicochemical properties, and accommodate diverse substituents within target- and pathogen-specific molecular designs [16,17,22]. These principles can guide the rational development of next-generation pyrrolidine-based antibacterial agents and resistance-modifying adjuvants [158,159].

## 4. Translational Challenges and Future Perspectives

### 4.1. Emerging Pyrrolidine-Based Antibacterial Candidates

Recent advances in antibacterial drug discovery have highlighted the pyrrolidine scaffold as a versatile heterocycle for the development of antibacterial agents and antibacterial adjuvants. The broad structural diversity of pyrrolidine-containing compounds, ranging from natural alkaloids and synthetic derivatives to hybrid molecules and beta-lactam enhancers, demonstrates the considerable adaptability of this heterocycle in medicinal chemistry. Several recently reported pyrrolidine-based compounds displayed significant activity against clinically relevant pathogens, including MDR strains of *Staphylococcus aureus*, *Pseudomonas aeruginosa*, and *Mycobacterium tuberculosis* [24,45,135,152,158,159].

Among the most promising compounds are hybrid molecules that combine the pyrrolidine nucleus with traditional antibacterial pharmacophores. Such an approach often results in compounds exhibiting improved target affinity, dual mechanisms of action, and reduced susceptibility to resistance mechanisms. Examples include oxadiazole–pyrrolidine hybrids, rifamycin–quinolone conjugates such as Rifaquizinone (TNP-2092), and pyrrolidine-containing chalcones, all of which illustrate the potential of the pyrrolidine scaffold to support the design of next-generation antibacterial candidates [135,136,156].

Particularly noteworthy is the appearance of pyrrolidine derivatives that act as resistance-modulating agents rather than as direct antibacterial compounds. Efflux pump inhibitors and beta-lactam enhancers, such as WCK-5153, exemplify a strategy to restore the efficacy of existing antibiotics against resistant pathogens [158,159]. Such compounds may represent an attractive solution for extending the clinical lifespan of currently available antibacterial therapies while reducing the need for entirely new antibiotic classes.

### 4.2. Challenges in the Development of Pyrrolidine-Based Antibacterial Agents

While numerous pyrrolidine derivatives have shown promising biological activity across diverse therapeutic areas, only a minority have advanced beyond preclinical assessment and early clinical investigation. Many studies remain restricted to in vitro antibacterial screening, making it difficult to assess the true therapeutic potential of the reported compounds [24,118,135,152].

Another important limitation concerns pharmacokinetic optimisation because structural modifications that improve antibacterial activity may adversely affect solubility, metabolic stability, or bioavailability [16,108,111,149]. Although the pyrrolidine ring frequently improves target binding, membrane permeability, and antibacterial potency, the incorporation of additional substituents may negatively influence metabolic stability, plasma protein binding, or bioavailability. Consequently, future drug development efforts must balance antibacterial efficacy with favourable ADMET properties [16,135,149,157].

The continuous evolution of bacterial resistance also remains a significant challenge. Even highly potent antibacterial candidates may eventually become vulnerable to target mutations, enzymatic degradation, biofilm formation, or efflux-mediated resistance. Therefore, the long-term success of pyrrolidine-derived antibacterials will likely depend on their ability to inhibit multiple resistance mechanisms simultaneously [140,152,158,159].

Additional challenges extend beyond antibacterial potency alone. Toxicity and selectivity data remain unavailable for many pyrrolidine derivatives, as numerous studies are limited to preliminary in vitro evaluations [118,143,145,153].

Also, long-term clinical utility may be compromised by the appearance of resistance mechanisms such as efflux-mediated resistance, target modification, and biofilm formation [38,140,158,159]. The development of structurally complex pyrrolidine-containing molecules, particularly hybrid compounds and stereochemically rich scaffolds, may also present manufacturing challenges during large-scale production [16,21,135]. The commercial limitations continue to hinder antibacterial drug development, as high research costs and relatively limited economic returns often reduce industrial investment in new antibacterial agents [4,127].

### 4.3. Future Directions in Pyrrolidine Antibacterial Research

Future research should focus on the rational design of pyrrolidine-based compounds that address current resistance mechanisms while maintaining favourable safety profiles. SAR studies indicate that strategic substitution of the pyrrolidine ring can significantly influence antibacterial potency, selectivity, and pharmacokinetic behaviour [16,135,149,152]. The increasing application of computational methods, including molecular docking, molecular dynamics simulations, and artificial intelligence-assisted drug design, is expected to accelerate the identification and optimisation of novel pyrrolidine-based leads [135,144,145,149]. Computational approaches should be used to prioritise compounds and generate mechanistic hypotheses, but predicted target interactions should be confirmed through biochemical, microbiological, and genetic experiments.

Particular attention should be paid to multitarget antibacterial agents, antibiotic adjuvants, and compounds that disrupt biofilm formation or bacterial quorum sensing. These approaches may prove especially valuable against ESKAPE pathogens, which continue to represent one of the greatest threats to global public health [119,135,158,159]. In addition, hybridisation strategies that combine the pyrrolidine scaffold with traditional antibacterial pharmacophores are expected to remain a productive area of investigation [135,136,156].

The accumulated evidence supports the pyrrolidine as a scaffold for antibacterial activity. Its favourable physicochemical properties, structural versatility, and ability to enhance interactions with diverse biological targets position pyrrolidine-containing molecules among the most promising candidates for the future development of antibacterial drugs and resistance-modifying agents [16,135,152,158,159].

## 5. Limitations of the Review

Although pyrrolidine-based heterocycles have shown promising antibacterial potential in the literature, several limitations remain.

First, the available literature is dominated by in vitro investigations, whereas in vivo efficacy studies, pharmacokinetic evaluations, and comprehensive toxicity assessments remain comparatively scarce. Consequently, the translational relevance of many reported compounds cannot yet be fully established.

Second, the studies included in this review exhibit substantial methodological heterogeneity with respect to bacterial strains, susceptibility-testing protocols, reference standards, and reported activity parameters, including MIC, MBC, and inhibition-zone measurements. Such variability limits direct comparisons between compounds and complicates the identification of robust SAR across different studies. Also, evidence from the patent literature should be interpreted cautiously, as experimental details are often less extensive than in peer-reviewed publications.

A further limitation arises from the predominantly descriptive nature of many reports. Although numerous pyrrolidine derivatives have demonstrated promising antibacterial activity, detailed mechanistic investigations, target-validation studies, and systematic SAR analyses are often lacking. This gap impedes a comprehensive understanding of the molecular features highlighting antibacterial efficacy, selectivity, and resistance-modifying activity.

Additionally, publication bias toward biologically active compounds may lead to underreporting of negative or inconclusive findings, potentially overestimating the overall promise of the pyrrolidine scaffold. Finally, because this review focuses specifically on pyrrolidine-containing antibacterial agents, it does not provide a comparative assessment against other emerging heterocyclic scaffolds that are also being actively explored in antibacterial drug discovery.

The present work is a narrative rather than a systematic review. Although multiple scientific databases and complementary search terms were used, the literature search may not have captured all relevant publications, particularly non-English-language articles, conference proceedings, patents, and other grey literature. In addition, no formal risk-of-bias or study-quality assessment was performed, and the heterogeneity of the available data precluded quantitative synthesis or meta-analysis.

All these limitations mark the need for standardised biological evaluation protocols, comprehensive pharmacological characterisation, and mechanism-based investigations to facilitate the rational development and clinical translation of pyrrolidine-derived antibacterial agents.

## 6. Materials and Methods

This work was conducted as a comprehensive narrative review of the literature on pyrrolidine-containing antibacterial compounds. Relevant publications were identified through targeted searches of Clarivate Web of Science Core Collection, ScienceDirect, PubMed, and Google Scholar, covering publications available up to June 2026. The selection of references was based on their relevance to the topic and their contribution to the understanding of pyrrolidine-based antibacterial agents, consistent with the methodology of a narrative review. Sources were included if they provided relevant information on the chemistry, antibacterial activity, mechanism of action, clinical use, resistance-modifying effects, or medicinal-chemistry development of pyrrolidine-containing compounds. For investigational candidates, preference was given to studies reporting original experimental antibacterial data. The literature search employed keywords such as “pyrrolidine,” “pyrrolidine derivatives,” “pyrrolidine-based compounds,” “substituted pyrrolidines,” “heterocycles,” “five-membered heterocycles,” and “nitrogen-containing heterocycles”; antibacterial-related terms, including “antibacterials” and “antibiotics.” Additional keywords, such as “biological activity,” “drug candidates,” “drug discovery,” “drug design,” “natural antibacterials,” and “natural antibiotics,” were also used to expand the search scope. From the retrieved literature, studies describing natural and synthetic pyrrolidine derivatives with experimentally confirmed antibacterial activity were selected for inclusion in this review. The patent literature was included when it provided unique structural or pharmacological information unavailable in peer-reviewed publications.

Whenever possible, preference was given to peer-reviewed publications reporting original experimental data, while review articles were primarily used to provide historical context and complementary background information. Recently published articles were specifically evaluated to ensure that the review reflects the most current developments in pyrrolidine-based antibacterial research.

Chemical structures were generated using Biovia Draw 2024 [161] and MarvinSketch 23.10 [162], while the corresponding IUPAC names of the compounds were obtained from the PubChem database [26].

## 7. Conclusions

The evidence analysed in this review demonstrates that pyrrolidine has evolved from a simple five-membered nitrogen heterocycle into a valuable medicinal chemistry scaffold with broad relevance for antibacterial drug discovery. Its unique structural and physicochemical properties, including conformational flexibility, stereochemical diversity, and favourable pharmacokinetic characteristics, contribute to enhanced interactions with diverse bacterial targets. The clinical success of several approved antibiotics containing a pyrrolidine moiety, such as carbapenems, cephalosporins, fluoroquinolones, lincosamides, streptogramins, and tetracyclines, highlights the therapeutic relevance of this scaffold. Thus, potent MIC values alone should not be interpreted as predictors of clinical success.

The compounds reviewed can be broadly classified into three complementary categories: clinically established antibiotics, investigational direct-acting antibacterial agents, and resistance-modifying compounds designed to restore or potentiate existing antibacterial therapies. Across these structurally diverse compound classes, medicinal chemistry optimisation consistently demonstrates that the contribution of the pyrrolidine scaffold depends strongly on its substitution pattern, stereochemistry, and integration within the overall molecular architecture rather than on the heterocycle alone.

Recent studies have demonstrated that natural pyrrolidine alkaloids, synthetic derivatives, hybrid molecules, and antibacterial adjuvants exhibit promising activity against clinically relevant and MDR pathogens. SAR investigations indicate that strategic modification of the pyrrolidine nucleus can significantly influence antibacterial potency, target selectivity, and resistance-modulating effects.

The accumulated evidence supports the pyrrolidine scaffold as a versatile platform for the development of next-generation antibacterial agents. Additional in vivo studies, pharmacokinetic evaluations, and mode-of-action studies are required to facilitate the translation of promising pyrrolidine-based candidates into clinically useful antibacterial therapies. Beyond the development of novel antibacterial agents, pyrrolidine-containing compounds are increasingly emerging as valuable components of resistance-modifying strategies, including β-lactam enhancers, efflux pump inhibitors, and antibiofilm adjuvants that may extend the clinical lifespan of existing antibiotics.

## Figures and Tables

**Table 1:** FDA-approved antibacterial agents containing a pyrrolidine or pyrrolidinium moiety (1996–2026).

No.	Antibacterial Compound	FDA Approval Year	Antibacterial Class (Generation)	References
1	Cefepime	1996	Beta-lactam cephalosporin (4th generation)	[28]
2	Meropenem	1996	Beta-lactam carbapenem	[28]
3	Ertapenem	2001	Beta-lactam carbapenem	[29]
4	Gemifloxacin	2003	Fluoroquinolone	[30]
5	Doripenem	2007	Beta-lactam carbapenem	[31]
7	Finafloxacin	2014	Fluoroquinolone	[32]
8	Eravacycline	2018	Tetracycline	[33]
9	Cefiderocol	2019	Beta-lactam cephalosporin (5th generation)	[34]
6	Ceftobiprole	2024	Beta-lactam cephalosporin (5th generation)	[35]
10	Cefepime + Zidebactam	2026	Beta-lactam cephalosporin (4th generation) + a beta-lactamase inhibitor and non-beta-lactam	[36,37]

**Table 2:** Classification of antibiotics incorporating a pyrrolidine moiety within their molecular structure, organised according to therapeutic class and subclass. (Ref. = references).

No.	Therapeutic Class	Subclass	Representatives	Ref.
1	Beta-lactam antibiotics	Carbapenemes	Doripenem	[53]
			Ertapenem	[53]
			Meropenem	[53]
			Panipenem	[54]
		Cephalosporins	Cefepime	[55]
			Cefiderocol	[56]
			Ceftobiprole	[57]
2	Fluoroquinolones	-	Clinafloxacin	[58]
			Finafloxacin	[58]
			Gemifloxacin	[58]
			Lascufloxacin	[58]
			Premafloxacin	[59]
			Sitafloxacin	[58]
			Trovafloxacin	[58]
3	Lincosamides	-	Lincomycin	[55]
			Clindamycin	[55]
4	Streptogramins	-	Quinupristin/Dalfopristin	[60]
5	Tetracyclines	-	Rolitetracycline	[61]
		Glycylcyclines	Eravacycline	[62]

## Data Availability

No new data were created or analyzed in this study. Data sharing is not applicable to this article.

## Abbreviations

The following abbreviations are used in this manuscript:

ABSSI	Acute Bacterial Skin And Skin Structure Infections
ACE	Angiotensin-Converting Enzyme
ADME	Absorption, Distribution, Metabolism, Excretion
CABP	Community-Acquired Bacterial Pneumonia
CFTR	Cystic Fibrosis Transmembrane Conductance Regulator
cIAIs	Complicated Intra-Abdominal Infections
CRE	Carbapenemase-Producing *Enterobacteriaceae*
CVB3	Group B Coxsackievirus Type 3
DHP-1	Dehydropeptidase I
DNA	Deoxyribonucleic Acid
DOS	Diversity-Oriented Synthesis
DPP-4	Dipeptidyl Peptidase-4
EMA	European Medicines Agency
ESBL	Extended-Spectrum Beta-Lactamase
ESKAPE	*Enterococcus faecium*, *Staphylococcus aureus*, *Klebsiella pneumoniae*, *Acinetobacter baumanii*, *Pseudomonas aeruginosa*, and *Enterobacter* sp.
FDA	Food And Drug Administration
Glc-N-6-P	Glucosamine-6-Phosphate Synthase
HCV	Hepatitis C Virus
KRAS	Kirsten Rat Sarcoma Viral Oncogene Homolog
MBC	Minimum Bactericidal Concentration
MIC	Minimal Inhibitory Concentration
MDR	Multidrug-Resistant
MRSA	Methicillin-Resistant *Staphylococcus Aureus*
MSSA	Methicillin-Susceptible *Staphylococcus Aureus*
NS3/4A	Non-Structural Proteins 3/4a
NS5A	Non-Structural Protein 5a
PBPs	Penicillin-Binding Proteins
PCAMs	Pyrrolidine Carboxamide Family
PMBN	Polymyxin B Nonapeptide
PDE-5	Phosphodiesterase Type 5
RNA	Ribonucleic Acid
SAB	*Staphylococcus Aureus* Bloodstream Infections (Bacteremia)
SAR	Structure-Activity Relationships
UTI	Urinary Tract Infections
VRE	Vancomycin-Resistant *Enterococcus faecium*
VRSA	Vancomycin-Resistant *Staphylococcus aureus*
WHO	World Health Organisation
